# Guanylate binding protein 5 is an immune‐related biomarker of oral squamous cell carcinoma: A retrospective prognostic study with bioinformatic analysis

**DOI:** 10.1002/cam4.7431

**Published:** 2024-07-08

**Authors:** Masayo Hasegawa, Yusuke Amano, Atsushi Kihara, Daisuke Matsubara, Noriyoshi Fukushima, Hideyuki Takahashi, Kazuaki Chikamatsu, Hiroshi Nishino, Yoshiyuki Mori, Naohiro Yoshida, Toshiro Niki

**Affiliations:** ^1^ Department of Integrative Pathology Jichi Medical University Shimotsuke Tochigi Japan; ^2^ Department of Otolaryngology‐Head and Neck Surgery Jichi Medical University Saitama Medical Center Saitama Japan; ^3^ Department of Pathology, Faculty of medicine University of Tsukuba Tsukuba Ibaraki Japan; ^4^ Department of Otolaryngology‐Head and Neck Surgery Gunma University Graduate School of Medicine Maebashi Gunma Japan; ^5^ Department of Otolaryngology‐Head and Neck Surgery Jichi Medical University Shimotsuke Tochigi Japan; ^6^ Department of Dentistry, Oral and Maxillofacial Surgery Jichi Medical University Saitama Medical Center Saitama Japan

**Keywords:** GBP5, OSCC, PD‐L1, stromal pattern

## Abstract

**Background:**

Cancer utilizes immunosuppressive mechanisms to create a tumor microenvironment favorable for its progression. The purpose of this study is to histologically characterize the immunological properties of the tumor microenvironment of oral squamous cell carcinoma (OSCC) and identify key molecules involved in the immunological microenvironment and patient prognosis.

**Methods:**

First, overlapping differentially expressed genes (DEGs) were screened from OSCC transcriptome data in public databases. Correlation analysis of DEGs with known immune‐related genes identified genes involved in the immune microenvironment of OSCC. Next, stromal patterns of tumor were classified and immunohistochemical staining was performed for immune cell markers (CD3, CD4, Foxp3, CD8, CD20, CD68, and CD163), programmed death‐ligand 1 (PD‐L1), and guanylate binding protein 5 (GBP5) in resected specimens obtained from 110 patients with OSCC who underwent resection. Correlations between each factor and their prognostic impact were analyzed.

**Results:**

Among the novel OSCC‐specific immune‐related genes screened (including *ADAMDEC1*, *CXCL9*, *CXCL13*, *DPT*, *GBP5*, *IDO1*, and *PLA2G7*), GBP5 was selected as the target gene. Histopathologic analysis showed that multiple T‐cell subsets and CD20‐positive cells were less common in the advanced stages, whereas CD163‐positive cells were more common in advanced stages. The immature type in the stromal pattern category was associated with less immune cell infiltration, lower expression of PD‐L1 in immune cells, lower expression of GBP5 in the stroma, and shorter overall survival and recurrence‐free survival. Expression of GBP5 in the tumor and stroma correlated with immune cell infiltration of tumors and PD‐L1 expression in tumor and immune cells. Patients with low tumor GBP5 expression and high stromal expression had significantly longer overall survival and recurrence‐free survival.

**Conclusions:**

The stromal pattern category may reflect both invasive and immunomodulatory potentials of cancer‐associated fibroblasts in OSCC. GBP5 has been suggested as a potential biomarker to predict the prognosis and therapeutic efficacy of immune checkpoint inhibitors.

## 1 INTRODUCTION

The oral cavity is the primary site of head and neck cancer, with approximately 370,000 new cases (2.0% of all cancers) and 170,000 deaths (1.8% of all cancers) reported annually worldwide.^1^ By histology, squamous cell carcinoma accounts for more than 90% of oral cancers.^2^ Oral squamous cell carcinoma (OSCC) is a highly invasive cancer characterized by high local recurrence and metastasis.^3^ Despite advances in multimodality treatments, such as surgery, chemotherapy, and radiation therapy, the 5‐year survival rate is approximately 50%–60%, with no improvement in treatment outcomes.^4^ In recent years, molecular‐targeted therapies have shown promise for the treatment of other types of cancers, and their efficacy has been improving. Typical target molecules, such as epidermal growth factor receptor (EGFR), human epidermal growth factor receptor type 2 (HER2), fibroblast growth factor receptor (FGFR), are mutated or amplified in only about 20% of head and neck cancers.^5^ To develop new therapeutic agents with improved efficacy, it is important to investigate target factors that are more selective for head and neck cancers.

Previous reports have shown that the tumor microenvironment, which is composed of the cellular components of the cancer parenchyma (cancer cells), stroma (including immune cells, vascular system cells, and fibroblasts), and non‐cellular components (including extracellular matrix), strongly influences malignant phenotypes, such as cancer invasion, metastasis, and drug resistance during cancer growth and development.^6^ In particular, it has been shown that during cancer progression, stromal cells interact with cancer cells through diverse molecular mechanisms mediated by various humoral factors such as cytokines, chemokines, and growth factors, resulting in the formation of an immunosuppressive microenvironment.^7^ Several studies have reported on combining several markers to assess the prognosis of OSCC. High expression of p53, EGFR, and cyclin A2 and low expression of p16 were related with decreased survival.^8^ Cytoplasmic and membranous expression of EGFR and overexpression of p53 were a poor prognostic marker in early stage OSCC.^9^ High co‐expression of podoplanin and matrix metalloproteinase‐9 was associated with poor prognosis.^10^ Overexpression of extracellular matrix metalloproteinase inducer (EMMPRIN) in OSCC was associated with high proliferative activity (Ki‐67 expression in more than 50% tumor cells) and poor prognosis.^11^

Since cancer cells are abnormal cells that have lost their self‐antigens, they are eliminated by immune surveillance mechanisms through innate or acquired immunity. However, if cancer cells are not eliminated for some reason, they escape from the host anti‐tumor immunity through the following mechanisms: (1) selective proliferation of only immune‐unresponsive cells among cancer cells, and (2) proliferation by utilizing immunosuppressive mechanisms.^12^ Several immune checkpoint inhibitors (ICIs) have been developed to inhibit immunosuppressive mechanisms. The anti‐programmed cell death‐protein 1 (PD‐1) inhibitors (pembrolizumab and nivolumab) have been approved by the US Food and Drug Administration (FDA) for head and neck squamous cell carcinoma.^13^ These drugs are now considered new treatment options for recurrent metastatic squamous cell carcinoma of the head and neck in Japan. However, the response rate to these drugs is limited to less than 20% and reliable biomarkers to predict the efficacy of these drugs are lacking.^14^, ^15^

Therefore, various studies are underway to elucidate the mechanisms of anti‐tumor immune responses and the immunosuppression of innate and acquired immunity in many cancer types. Tumor infiltrating lymphocytes (TILs) have been evaluated in many cancers and are known that TILs play an important role in cancer progression.^16^ The expression of tumor‐infiltrating immune cells and immune checkpoint molecules in tumors or immune cells has also been discussed as a potential biomarker for prognosis and predicting of the therapeutic response to immunotherapy.^17^, ^18^ Furthermore, it has been reported that cancer‐associated fibroblasts (CAFs), non‐immune cells that reside in the cancer stroma, influence cancer and immune cells and are involved in tumor growth, metastasis, and malignant transformation.^19^ Our laboratory previously proposed the concept of stromal pattern (SP) as a histological indicator of desmoplastic reaction (DR) in OSCC, which reflects the function of CAFs, and reported that prognosis is influenced by differences in SP.^20^

However, there is no consensus regarding previous report findings on the impact of tumor‐infiltrating immune cells and the expression of immune checkpoint molecules on patient prognosis and drug response. In addition, many studies have focused on specific immune cell lineages, such as T‐cell and macrophage lineages, in the tumor microenvironment. There is a paucity of studies that have comprehensively examined the behavior of immune cells of multiple lineages in the microenvironment.^21^ Furthermore, there are few studies on the interactions between immune cells and CAFs in the tumor microenvironment, particularly regarding the molecular mechanisms underlying their regulation in OSCC.^22^

The aim of this study was to elucidate using histopathological analysis the immunological properties of the OSCC tumor microenvironment and identify new immune‐related genes regulating them. We believe that a systematic understanding of the tumor immune microenvironment, including interactions between cancer and stromal cells, will lead to the identification of effective biomarkers for predicting patient prognosis and response to drugs and of future stratified therapies.

## 2 MATERIALS AND METHODS

### 2.1 Data collection, screening, and identification of differential expressed genes (DEGs)

We downloaded the mRNA sequence data of patients with head and neck squamous cell carcinoma (HNSCC) (Illumina Hiseq RNAseq V2, RSEM raw and normalized data) and clinical information in The Cancer Genome Atlas (TCGA) database from the FireBrowse website (https://firebrowse.org/, accessed October 31, 2022). The data included 556 HNSCC and 44 normal samples. Among the HNSCC samples, those with primary lesions on the tongue, base of tongue, floor of mouth, buccal mucosa, hard palate, alveolar crest, lips, and oral cavity were included, and those with case as the normal samples were excluded. A total of 319 OSCC and 44 normal samples were used in this analysis. The TCC package (version 1.42.0) of R (The R Foundation for Statistical Computing, Vienna, Austria, version 4.2.2) was used to identify DEGs. mRNA expression levels and clinical information from the GSE30784 dataset were downloaded from the GEO database (accessed November 11, 2022, https://www.ncbi.nlm.nih.gov/geo/). The data included 167 OSCC and 45 normal samples obtained from independent controls. The GEO 2R online tool (https://www.ncbi.nlm.nih.gov/geo/geo2r/, R version 3.2.3, Limma package version 3.26.8) was used. In these database analyses, the criteria for DEGs were |log_2_ fold change (FC) | >2 and false discovery rate (FDR) <0.05. Venn diagrams were created using the Venn package (version 1.7.3) in R software (version 4.2.2), to identify overlapping DEGs in the TCGA and GSE30784 datasets.

### 2.2 Correlation analysis of DEGs

Normalized mRNA expression data obtained from TCGA were log_2_ transformed and used in subsequent studies. Correlation analysis was performed for the mRNA expression levels of known immune cell‐associated genes and DEGs that overlapped in TCGA and GSE30784 datasets. The following genes were selected as immune cell‐related markers according to previous reports: macrophages (CD68, CD163, mannose receptor C‐type 1 [MRC1], CD14, colony‐stimulating factor 1 [CSF1], CSF2); T cells [CD3E]; cytotoxic T cells (CTL: CD8A, interferon‐gamma [IFNγ], granzyme B [GZMB]); regulatory T cells (Treg: CD4, forkhead box P3 [Foxp3]); B cells (CD19); immunosuppressive (IL10, transforming growth factor beta 1 [TGFB1]); and proinflammatory (interleukin [IL]6, IL8).^23^ Pearson's correlation coefficient was calculated to calculate the correlation coefficient (*r*), and |*r*| >0.4 was defined as a significant correlation (*p* < 0.05). The correlation matrix was constructed using the corrplot package (version 0.92) in R software.

### 2.3 Patient cohort and tissue specimens

This retrospective study included 110 patients with primary OSCC who underwent surgical resection (61 male; 49 female; mean and median ages, 63.4 and 66.0 years, respectively) at the Jichi Medical University Hospital between 2010 and 2021. Patients were excluded from the analysis if they had a history of preoperative chemotherapy or radiation therapy, recurrence, preoperatively evident metastasis, small resection specimens (including biopsy specimens), or prognostic follow‐up data were unavailable. Resection specimens from eligible patients were formalin‐fixed paraffin‐embedded (FFPE) and used for histopathologic examination. Patient clinical information was obtained by reviewing medical records.

**FIGURE 1:**
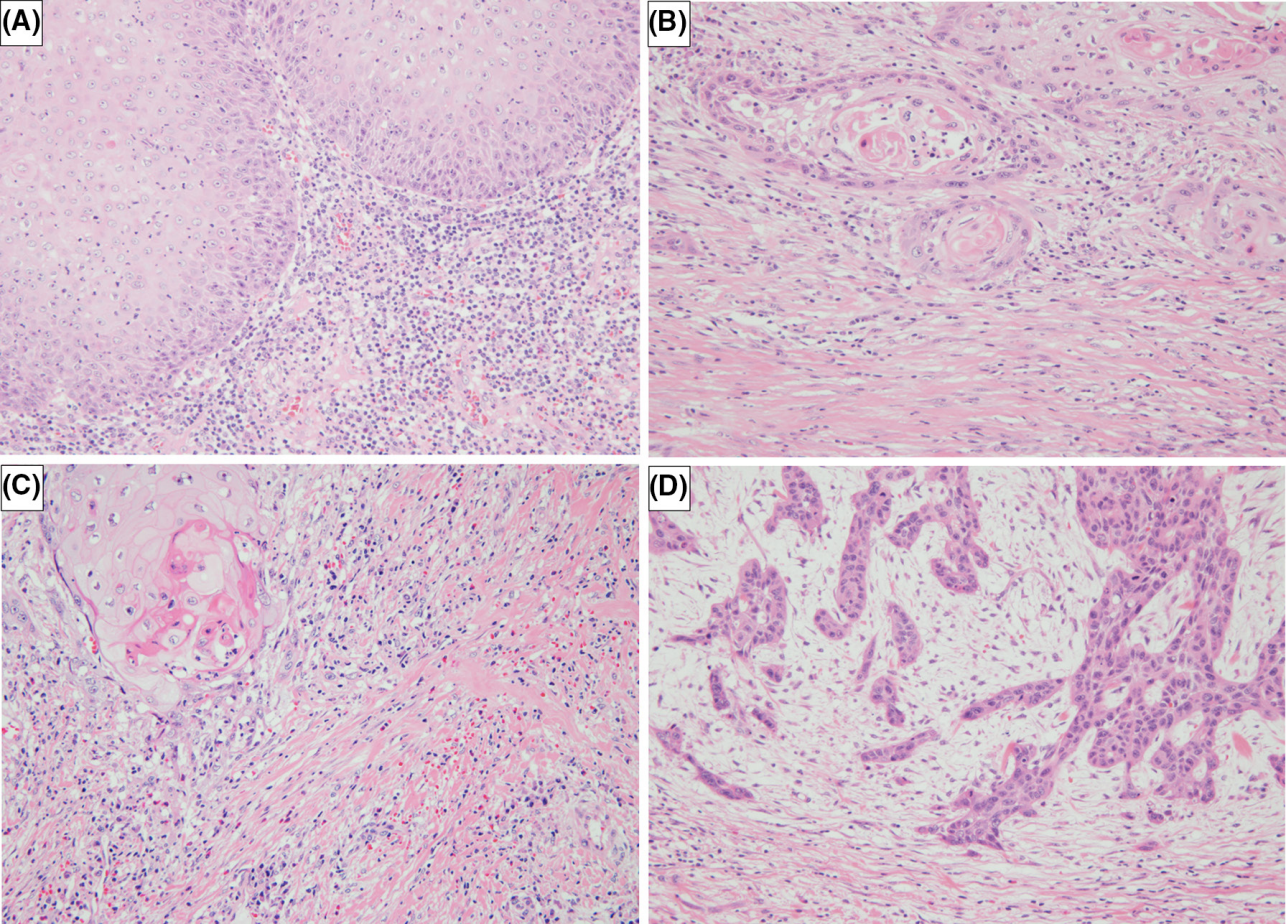
Representative figures of stromal pattern by HE. Original magnification × 200. (A) The inflammatory type is characterized by an abundant lymphocytic infiltration without an obvious desmoplastic reaction. (B) The mature type shows fibrous stroma without keloid‐like collagen or a myxoid stroma. (C) The intermediate type is characterized by the presence of keloid‐like collagen. (D) The immature type includes a myxoid stroma within abundant amorphous extracellular matrix material. HE, hematoxylin–eosin.

All hematoxylin–eosin‐stained FFPE tissue specimens were examined under an optical microscope (Olympus BX53F2, Tokyo, Japan). Histopathologic diagnosis of the tumors was based on the WHO Classification of Head and Neck Tumors, 5th edition (2022).^24^ The following parameters were used to determine the histopathologic diagnosis: degree of differentiation (Grade); depth (T classification); regional lymph node involvement (N classification); depth of invasion (DOI); and presence of vascular and peripheral nerve invasion (lymphatic invasion [Ly], vascular invasion [V], perineural invasion [Neu]), were evaluated according to the American Joint Committee on Cancer/International Union Against Cancer (AJCC/UICC) classification of TNM malignancies, 8th edition.^25^ The morphologic pattern of invasion was classified by the method that was previously described as Yamamoto‐Kohama classification.^26^ In addition, we evaluated the SP observed at the invasive front in the deepest specimens, using a previously published method.^20^ SPs are classified into four types: inflammatory, mature, intermediate, and immature.^20^ In inflammatory type, no DR was evident in the stroma and a diffuse inflammatory cell infiltrate was observed. In mature type, mature collagen fibers were seen without keloid‐like collagen or myxoid stroma. In intermediate type, eosinophilic keloid‐like collagen was mixed with mature collagen fibers. In immature type, myxoid stroma was found with a basophilic amorphous extracellular matrix (Figure 1).

### 2.4 Immunohistochemistry (IHC)

Immunohistochemical staining was performed using an automated staining system, the Ventana Discovery XT (Roche, Basel, Switzerland). The deepest tumor block of the primary lesion was selected; 4‐μm thin sections were prepared from an FFPE tissue, deparaffinized, and heat‐treated (60 min) in cell conditioning 1 (CC1) buffer (pH 8.5 Ventana Medical Systems, Tucson, AZ, USA) for antigen retrieval, followed by a primary antibody reaction. The primary antibodies in this study were CD3, CD4, Foxp3, CD8, CD20, CD68, CD163, PD‐L1, and guanylate binding protein 5 (GBP5). The DAB Map Detection Kit (Ventana) was used for antigen detection, and the Amplification Kit (Roche) was used for sensitizing reagents. All sections were contrast stained with hematoxylin. Lymph nodes were used as positive controls for all the markers. A summary of the primary antibodies and staining conditions for the immunohistological staining, is presented in Table 1.

**TABLE 1:** Antibodies used in the present study.

Antibody	Source	Host	Clone	Dilution (×)	Amplification Kit
CD3	Leica Biosystems, Newcastle, UK	Mouse monoclonal	LN10	200	+
CD4	Abcam, Cambridge, UK	Rabbit monoclonal	SP35	25	+
Foxp3	Cell Signaling, Danvers, Massachusetts, USA	Rabbit monoclonal	D2W8E	25	+
CD8	Abcam, Cambridge, UK	Mouse monoclonal	C8/144B	25	+
CD20	Cell Signaling, Danvers, Massachusetts, USA	Mouse monoclonal	L26	500	+
CD68	Dako/Agilent, Santa Clara, CA, USA	Mouse monoclonal	KP1	2000	−
CD163	Leica Biosystems, Newcastle, UK	Mouse monoclonal	10D6	2000	−
GBP5	Proteintech, Chicago, IL, USA	Rabbit polyclonal	ー	500	+
PD‐L1	Abcam, Cambridge, UK	Rabbit monoclonal	28–8	100	+

Abbreviations: GBP5, guanylate binding protein 5; PD‐L1, programmed death‐ligand 1.

### 2.5 Evaluation of IHC

Necrosis, ulceration, and abscess areas were excluded from the evaluation of all immunostained specimens.

To count the tumor‐infiltrating immune cells, the entire specimen was first observed under low magnification, and then five representative fields of view were selected under a high magnification of 200× in the area containing the tumor and stroma. Images of the selected fields of view were captured by the Olympus cellSens software version 4.1 (Olympus, Tokyo, Japan) and saved as Tag Image File Format files. The images were imported into Image J software (ver1.54d/Java 1.8.0). The positive cells per unit area (3.70 × 10^5^ μm^2^) were counted manually. The median number of positive cells was used as the cutoff for classifying the cases into two groups.

PD‐L1 is expressed in the cytoplasm and cell membranes of tumor cells (TC), and immune cells (IC: macrophages and lymphocytes), regardless of the intensity of expression. The percentage of PD‐L1 expression in TC and IC, defined as TC or IC values >5%, were judged to be PD‐L1 positive.

The expression of GBP5 was scored semi‐quantitatively based on the staining intensity and extent of cancer and stromal cells, as previously reported.^27^, ^28^ The staining intensity scores were categorized as negative (0), mild (1), moderate (2), strong (3), while the staining extent as <5% (0), 5 to <25% (1), 25 to <50% (2), 50% to <75% (3), ≥ 75% (4). The total intensity and extent of staining scores in tumor and stromal cells were defined as the GBP5 tumor and GBP5 stromal scores (ranging from 0 to 7), respectively. The median score was used as the cutoff for categorizing the patients into High and Low groups.

The IHC evaluation was performed independently by two histopathologists (MH and YA) who were blinded to the relevant clinical information. If the difference in the number of positive immune cells counted by the two evaluators was <20%, the mean value was adopted as the positive cell count. On the other hand, if the difference was ≥20%, the evaluators discussed the reasons for the difference and repeated the evaluation until the difference was <20%. When the PD‐L1 and GBP5 evaluations disagreed between evaluators, the immunostained specimens were viewed together under a two‐headed microscope, and the agreed results were adopted.

### 2.6 Statistical analysis

All statistical analyses were performed using the R software or EZR (Saitama Medical Center, Jichi Medical University, Saitama, Japan, version 1.61).^29^

The normality of GBP5 score and number of tumor‐infiltrating immune cells was tested using the Shapiro–Wilk Normality test. Student's *t*‐test was used for comparison of continuous variables between two groups. One‐way analysis of variance was used to analyze the difference of continuous variables between three groups, and significant differences were tested using Bonferroni's multiple comparisons. Fisher's exact test was used to analyze nominal variables. Pearson's correlation analysis was performed to test the correlation between two continuous variables.

The endpoints of this study were overall survival (OS) and recurrence‐free survival (RFS), with OS defined as the time from the date of surgery to all‐cause mortality and RFS as the time from the date of surgery to recurrence and all‐cause mortality. Survival rates were calculated using the Kaplan–Meier method, and log‐rank tests were used to compare survival curves. Hazard ratios and 95% confidence intervals were calculated using the Cox proportional hazards model. All tests were based on two‐sided statistical analyses, and statistical significance was set at *p* < 0.05.

The study protocol was approved by the Ethics Review Committee of Jichi Medical University (Approval No: Rin 22‐012). As this was a retrospective study without additional risks to eligible patients, informed consent was obtained using the opt‐out method.

## 3 RESULTS

### 3.1 Screened and identified DEGs

**FIGURE 2:**
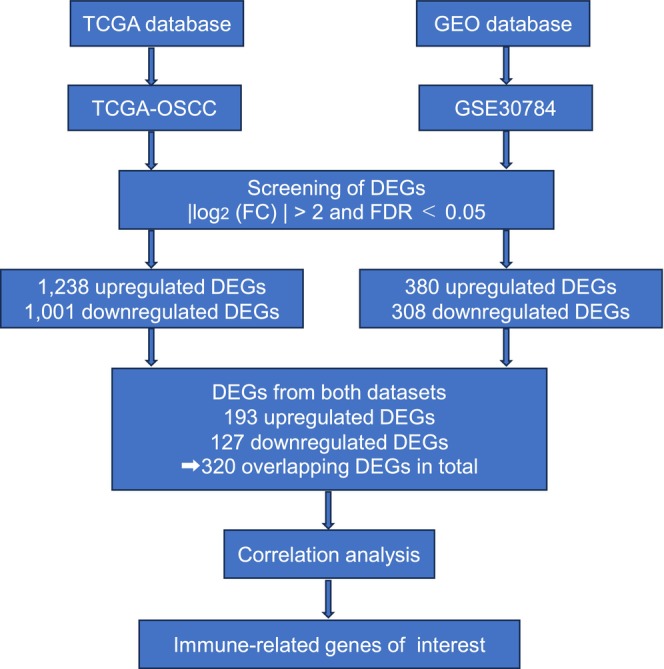
Flowchart for this study using bioinformatics data from TCGA and GEO. TCGA, The Cancer Genome Atlas; GEO, Gene Expression Omnibus; DEGs, differentially expressed genes; FC, fold change; FDR, false discovery rate.

**FIGURE 3:**
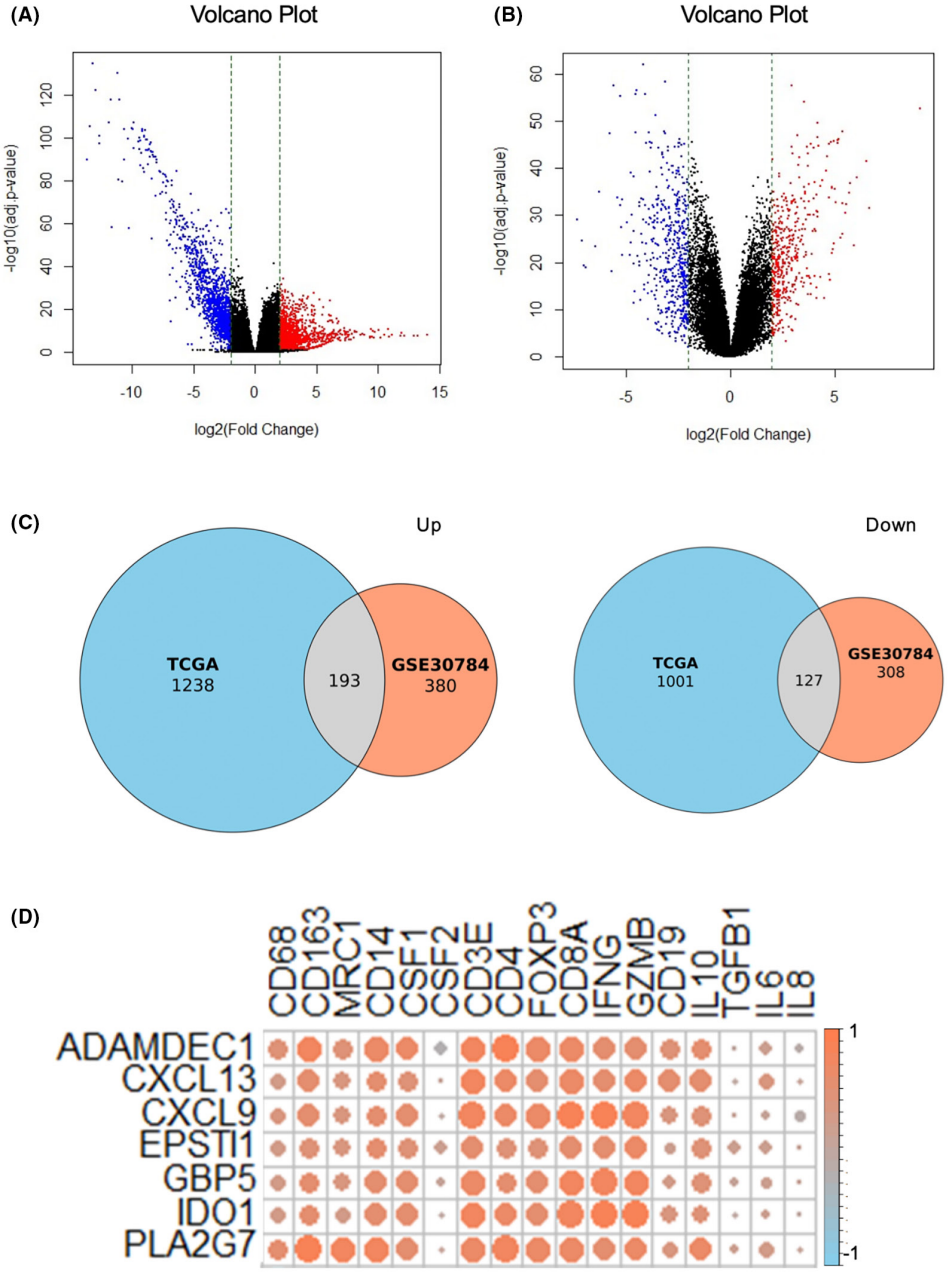
Identification of novel immune‐related genes by two database analysis. (A) Volcanic plots of 2239 DEGs (including 1238 upregulated genes and 1001 downregulated genes) from TCGA. (B) Volcanic plots of 688 DEGs (including 380 upregulated genes and 308 downregulated genes) from GEO. The red and blue spots indicate up and downregulated genes, respectively. (C) Venn diagrams show the overlapping DEGs from GEO and TCGA (Left: Upregulated DEGs, Right: Downregulated DEGs). Blue area: TCGA dataset; orange area: GEO dataset; cross area: Overlapping DEGs expressed in both databases. (D) Correlation matrix between DEGs overlapping the two datasets and known immune‐related genes. Red circles indicate positive correlation, blue circles indicate negative correlation. The size of the circle shows the strength of the correlation. TCGA, The Cancer Genome Atlas; GEO, Gene Expression Omnibus; DEGs, differentially expressed genes.

The workflow of this study bioinformatics analysis using public databases is shown in Figure 2. RNA sequencing data from TCGA‐OSCC, including 319 OSCC and 44 independent normal samples used to screen for 1238 upregulated DEGs and 1001 downregulated DEGs. Microarray data from the GSE30784 dataset containing 167 OSCC and 45 independent normal samples were also used to screen for 380 upregulated and 308 downregulated DEGs. Volcano plots were constructed to show the DEGs between OSCC and normal samples in each dataset (Figure 3A,B). Venn diagrams were plotted to identify overlapping DEGs in the TCGA and GEO datasets. We identified 320 overlapping DEGs, including 127 upregulated and 193 downregulated DEGs (Figure 3C).

### 3.2 Identification of novel immune‐related genes

We performed a correlation analysis of mRNA expressions between the identified overlapping DEGs and known immune‐related genes using TCGA‐OSCC data. We searched for DEGs that were significantly correlated with immune cell‐related markers, including macrophages (CD68, CD163, MRC1, CD14, CSF1, and CSF2); T cells (CD3E); Tregs (CD4 and Foxp3); and immune suppressor system (IL10 and TGFB1), as previously described. Based on this analysis, ADAM like decysin 1 (ADAMDEC1), C‐X‐C motif chemokine ligand 9 (CXCL9), CXCL13, dermatopontin (DPT), GBP5, indoleamine 2,3‐dioxygenase 1 (IDO1), and phospholipase A2 group VII (PLA2G7) were identified as novel immune‐related genes specific to OSCC (Figure 3D).

In a preliminary IHC study with a small number of OSCC cases, GBP5 was found to be more strongly expressed in OSCC than in the normal oral epithelium, suggesting that GBP5 is associated with tumor‐infiltrating immune cells. Therefore, we decided to focus on subsequent studies on GBP5.

### 3.3 Association between tumor‐infiltrating immune cells and clinicopathological factors

We counted the number of positive cells for each type of tumor‐infiltrating immune cell as described in the Methods section. Representative images of each immune cell subtype obtained by immunohistochemical staining and a summary of the numbers of positive immune cells are shown in Figure S1 and Table S1.

The association between tumor‐infiltrating immune cells and clinicopathological factors is summarized as follows: greater numbers of multiple T‐cell subtypes and CD20‐positive cells were associated with larger tumor size, earlier pathological stage, perineural invasion, and greater depth of invasion (*p* < 0.05). A greater number of CD163‐positive cells was associated with advanced pathological stages (*p* < 0.01). The number of cells positive for all immune cell subsets was significantly lower in the immature type than that in the inflammatory type. In addition, the numbers of positive cells for CD3, CD4, and CD8 were significantly lower in the mature/intermediate types than in the inflammatory type (Table S2).

### 3.4 PD‐L1 expression in OSCC tumor and immune cells

The PD‐L1 expressions in TC or IC were evaluated as described above, with the TC >5% or IC >5% criteria considered PD‐L1 positive (Figure S2). Of the 110 patients with OSCC, 20.0% (22/110) and 43.6% (48/110) were TC and IC positive, respectively.

TC‐negative and IC‐negative cases were the most common (50.9% [56/110]), followed by TC‐negative and IC‐positive cases (29.1% [32/110]), TC‐positive and IC‐positive cases (14.5% [16/110]), and TC‐positive and IC‐negative cases (5.5% [6/110]).

The associations between PD‐L1 expression and clinicopathological factors are shown in Table S3. The proportion of immature types was significantly lower in IC‐positive than in IC‐negative cases.

### 3.5 Expression of GBP5 in tumors and stroma in OSCC

**FIGURE 4:**
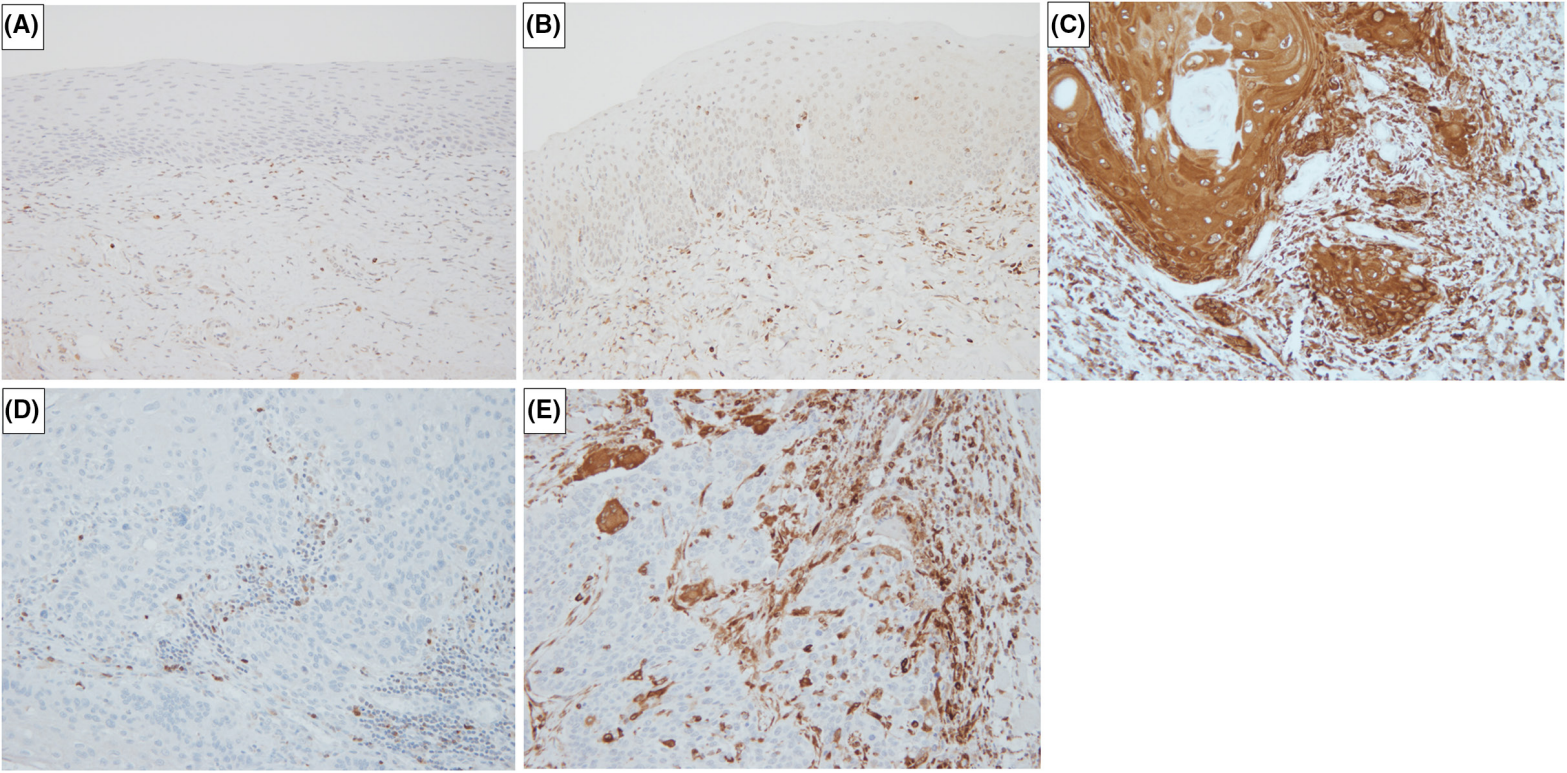
Representative immunohistochemical images of GBP5 in OSCC, normal oral epithelial tissues, and OED (original magnification: ×200). (A) Lack of expression of GBP5 in normal epithelium and weak expression in stroma. (B) Weak expression of GBP5 in tumor cells and stromal cells of OED. (C) Strong expression of GBP5 in tumor cells and stromal cells of OSCC. (D) Weak expression of GBP5 in tumor cells and stromal cells of OSCC. (E) Weak expression of GBP5 in tumor cells and strong expression in stromal cells of OSCC. GBP5, guanylate binding protein 5; OSCC, oral squamous cell carcinoma; OED, oral epithelial dysplasia.

GBP5 was absent or weakly expressed in the normal oral epithelium and oral epithelial dysplasia (OED) adjacent to OSCC. GBP5 was weakly to strongly expressed in the cytoplasm and cell membrane of OSCC and stromal cells, including immune cells and fibroblasts (Figure 4).

The mean score of the GBP5 tumor was 4.15 ± 1.72 with a median score of 4, and that of the GBP5 stroma was 5.71 ± 1.17 with a median score of 6. After analysis based on groups divided according to the median GBP5 tumor score of 0–4 (Low group) and 5–7 (High group); and those with a GBP5 stromal score of 0–5 (Low group) and 6–7 (High group), a significant positive correlation was observed between GBP5 tumor and GBP5 stromal scores (*r* = 0.607, *p* < 0.001).

The correlations between GBP5 expression and clinicopathological parameters are shown in Table 2. The GBP5 tumor score was significantly higher in the older group (*p* = 0.030). The GBP5 stromal score was associated with tumor size (*p* < 0.001), nodal metastasis (*p* = 0.024), pathological stage (*p* = 0.023), vascular invasion (*p* = 0.020), and depth of invasion (*p* = 0.003). The GBP5 stromal scores were significantly lower in the immature type than in the other types (*p* = 0.007).

**TABLE 2:** Correlations between GBP5 expression and clinicopathological parameters.

Parameters	GBP5 tumor		GBP5 stroma	
	(score, mean)	*p*	(score, mean)	*p*
Sex				
Male	4.16	0.951	5.57	0.176
Female	4.14		5.88	
Age (years)				
<65	3.74	0.030*	5.49	0.088
≧65	4.46		5.87	
Location				
Tongue	4.02	0.164	5.63	0.195
Others	4.56		5.96	
pT status				
Tis–2	4.29	0.187	5.94	0.001***
3–4	3.80		5.10	
pN status				
0	4.18	0.840	5.86	0.024*
1–3	4.10		5.30	
Grade				
1	4.25	0.453	5.81	0.314
2–3	4.00		5.56	
pStage				
0–II	4.16	0.983	5.90	0.023*
III–IV	4.15		5.38	
YK				
1–3	4.40	0.337	5.88	0.236
4	4.08		5.62	
Ly invasion				
Negative	4.23	0.553	5.87	0.053
Positive	4.03		5.43	
V invasion				
Negative	4.19	0.829	5.98	0.020*
Positive	4.12		5.47	
Neu invasion				
Negative	4.15	0.944	5.76	0.506
Positive	4.17		5.60	
Depth				
<10	4.31	0.438	5.92	0.003**
≧10	4.00		5.17	
SP				
Inf/Mat/Int	4.35	0.269	5.96	0.007**
Imm	3.93		5.15	

Abbreviations: GBP5, guanylate binding proteinImm, immature; Inf/Mat/Int, inflammatory/mature/intermediate; SP, stromal pattern; YK, Yamamoto–Kohama.

* *p* < 0.05.

** *p* < 0.01.

*** *p* < 0.001.

### 3.6 Correlation of tumor infiltrating immune cells with PD‐L1 expression

We analyzed the correlation between tumor‐infiltrating immune cells and PD‐L1 expression. TC‐positive cases had significantly more CD3‐, CD4‐, CD8‐, CD68‐, and CD163‐positive cells than the TC‐negative cases. IC‐positive cases had significantly more positive cells in all immune cell subsets than the IC‐negative cases (Table S4).

### 3.7 Correlation of tumor infiltrating immune cells with GBP5 expression

Both the high GBP5 tumor and GBP5 stromal score groups had more tumor‐infiltrating immune cells in all subtypes than the respective low groups (Table 3).

**TABLE 3:** Correlations of tumor‐infiltrating immune cells with GBP5 expression in patients with OSCC.

Marker	GBP5 tumor score			GBP5 stroma score		
	High *n* (%)	Low *n* (%)		High *n* (%)	Low *n* (%)	
	52 (47.27)	58 (52.73)	*p*	68 (61.82)	42 (38.18)	*p*
CD3	723.88	410.41	8.51e‐8	688.49	348.31	7.13e‐11
CD4	325.53	211.53	4.56e‐6	323.09	172.07	8.72e‐10
Foxp3	113.89	77.94	5.45e‐4	120.16	54.08	3.49e‐13
CD8	366.68	181.06	9.91e‐10	333.17	164.60	2.53e‐7
CD20	101.25	56.75	0.003	100.55	40.92	5.31e‐6
CD68	166.68	111.86	2.63e‐5	156.19	107.98	3.74e‐4
CD163	113.43	72.11	3.28e‐5	101.52	75.66	0.011

Abbreviations: GBP5, guanylate binding protein 5; OSCC, oral squamous cell carcinoma.

### 3.8 Correlation of PD‐L1 expression with GBP5 expression

**FIGURE 5:**
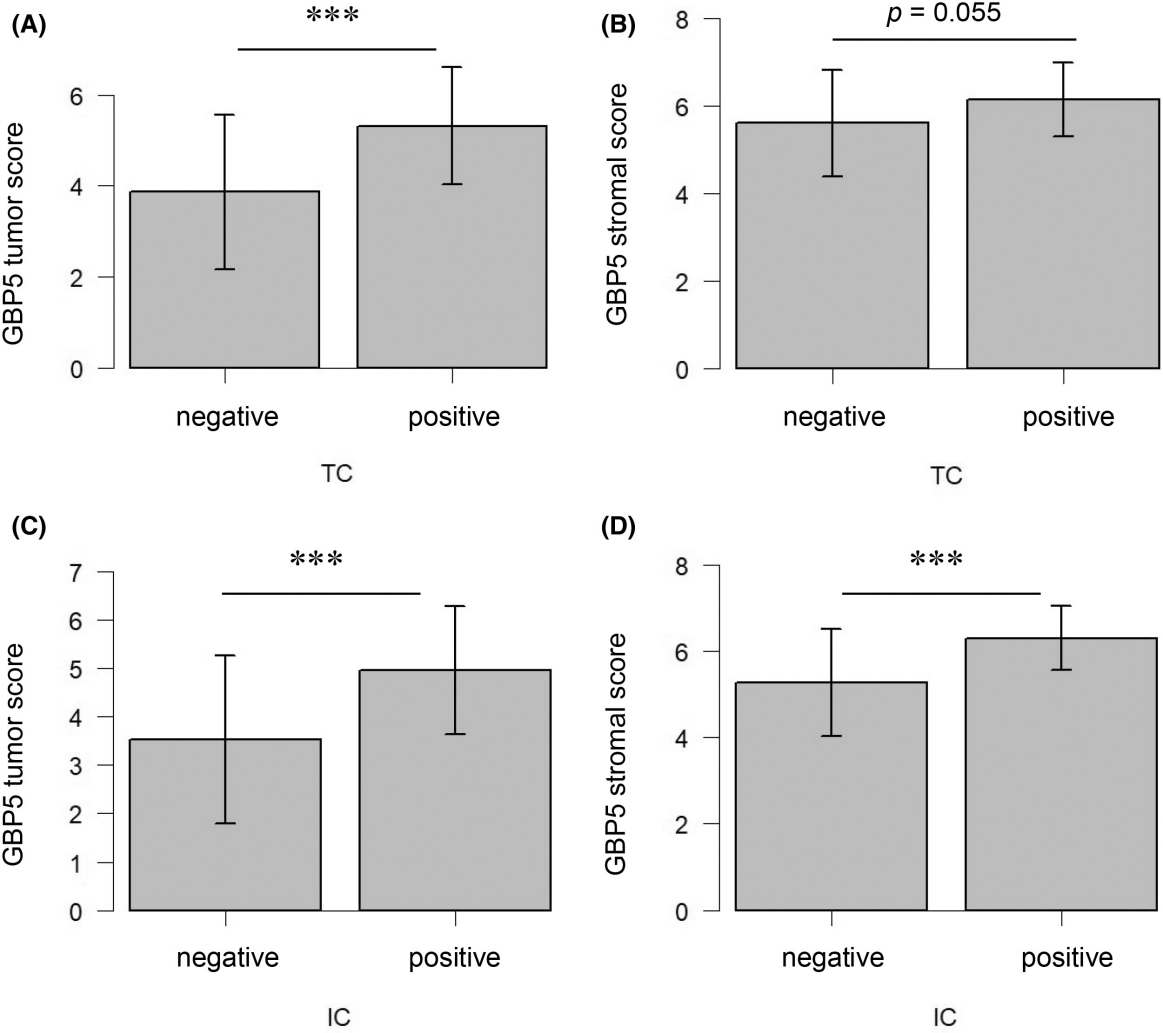
Correlation of PD‐L1 expression in tumor cells and immune cells with GBP5 expression. (A) GBP5 tumor scores grouped by TC status. (B) GBP5 stromal scores grouped by TC status. (C) GBP5 tumor scores grouped by IC status. (D) GBP5 stromal scores grouped by IC status. ***p < 0.001. PD‐L1, programmed death‐ligand 1; GBP5, guanylate binding protein 5; TC, PD‐L1 expression in tumor cells; IC, PD‐L1 expression in immune cells.

GBP5 tumor scores were higher in TC‐positive cases than in TC‐negative cases (*p* < 0.001). GBP5 stromal scores were also higher in TC‐positive cases than in TC‐negative cases, but the difference was less significant. Both scores were significantly higher in IC‐positive cases than in IC‐negative cases (*p* < 0.001) (Figure 5).

### 3.9 Survival analysis

#### 3.9.1 Survival analysis for all OSCC cohorts

The follow‐up period ranged from 16 days to 143.5 months, with a mean of 60.7 months and a median of 61.5 months. By the end of the follow‐up period, 32 patients (29.1%) developed recurrence and 22 (20.0%) had died. The 5‐year OS and RFS were 80.8% and 63.4%, respectively (Figure S3). A comparison of survival by clinicopathological factors showed that several factors were considered to have a prognostic impact and the SP categories were associated with OS and RFS (Table S5).

#### 3.9.2 Prognostic impact of tumor‐infiltrating immune cells

Next, we assessed the association between tumor‐infiltrating immune cells and patient prognosis. Kaplan–Meier curves showed that high numbers of CD68‐positive cells were significantly associated with longer RFS (*p* = 0.034). There was no significant association between the other immune cell subsets and patient survival (Table S6).

#### 3.9.3 Correlation between PD‐L1 expression and the patient prognosis

In addition, we investigated the effect of PD‐L1 expression in tumor and immune cells on patient prognosis. Regarding OS, there was no significant difference between PD‐L1 expression and prognosis for either TC or IC criteria (Figure S4a,b). TC criterion was no significantly associated with RFS (Figure S4c), whereas IC‐positive cases were associated with longer RFS (*p* = 0.042, hazard ratios [HR] 0.508, 95% confidence intervals [95% CI] 0.26–0.99) (Figure S4d).

#### 3.9.4 Prognostic role of GBP5 expression in the outcome of patients with OSCC

**FIGURE 6:**
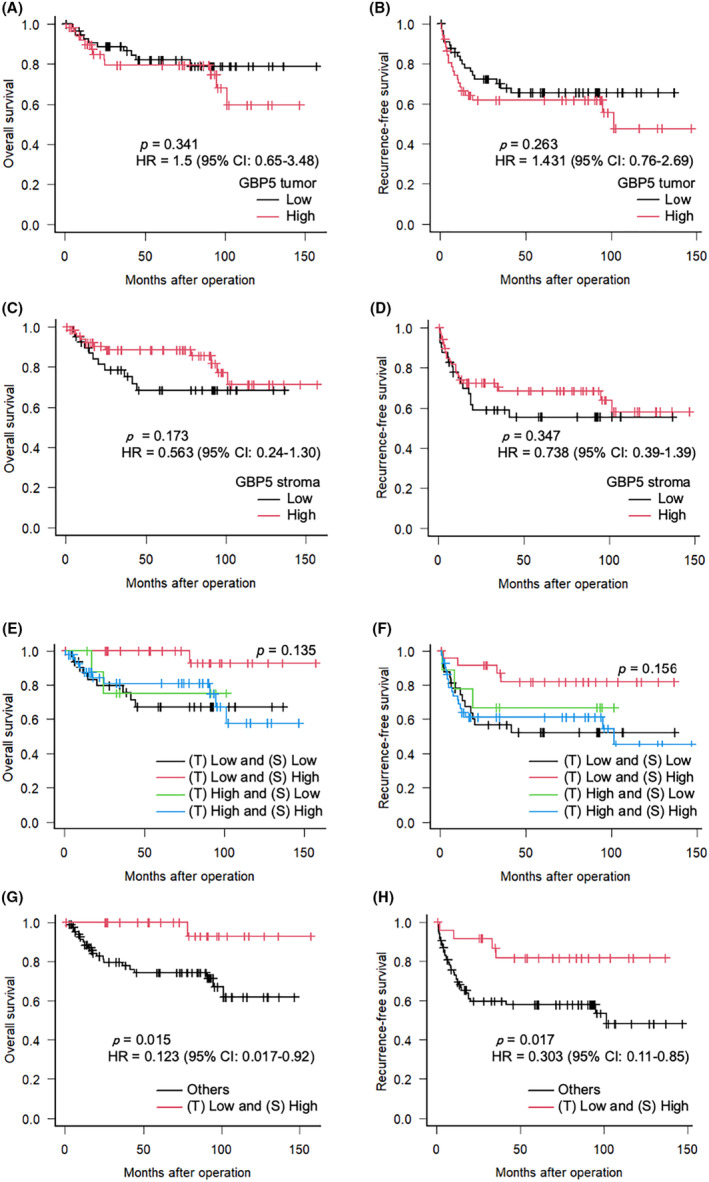
Kaplan–Meier curves showing the (A, C, E, G) overall and (B, D, F, H) recurrence‐free survival of patients with OSCC according to GBP5 expression. (E–H) Kaplan–Meier curves showing a comparison of survival rates according to a combination of GBP5 tumor and GBP5 stromal scores. (A–D, G, H) HR and 95% CI were indicated in the figures. *p*‐values were determined using log‐rank tests. OSCC, oral squamous cell carcinoma; GBP5, guanylate binding protein 5; HR, hazard ratios; CI, confidence intervals.

According to the Low and High groups that were based on the median GBP5 tumor and GBP5 stromal scores, there was no significant prognostic association in GBP5 tumor score (Figure 6A,B), and no significant differences in OS or RFS in GBP5 stromal score (Figure 6C,D) between the two groups.

Therefore, we performed a hierarchical univariate Kaplan–Meier analysis using GBP5 tumor and GBP5 stromal scores. The 5‐year survival rates were 100.0% in the GBP5 tumor (T) Low and GBP5 stroma (S) High group; followed by GBP5 (T) High and GBP5 (S) High group (80.8%); GBP (T) High and GBP5 (S) Low group (75.0%); and GBP5 (T) Low and GBP5 (S) Low group (67.1%). However, there was no significant difference in OS between the four groups (*p* = 0.135) (Figure 6E). Similarly, RFS at 5 years after surgery was 81.7% in the GBP5 (T) Low and GBP5 (S) High group; 66.7% in the GBP5 (T) High and GBP5 (S) Low group; 61.1% in the GBP5 (T) High and GBP5 (S) High group; and 52.3% in the GBP5 (T) Low and GBP5 (S) Low group; however, there was no significant difference among the four groups (*p* = 0.156) (Figure 6F). After a further division of the patients into two groups: GBP5 (T) Low and GBP5 (S) High, and the other groups, the GBP5 (T) Low and GBP5 (S) High expression groups were significantly associated with longer OS (*p* = 0.015, HR 0.123, 95% CI 0.017–0.92) and RFS (*p* = 0.017, HR 0.303, 95% CI 0.11–0.85) than the other groups (Figure 6G,H).

## 4 DISCUSSION

During cancer progression, various immune escape mechanisms are intricately involved and determine the immunological properties of the tumor microenvironment.^13^ This study aimed to histologically evaluate the immunological features of OSCC and identify the genes or molecules that regulate them and are associated with therapeutic efficacy and prognosis. This study suggested that GBP5 is associated with PD‐L1 expression and immune cell infiltration into tumors, which may also affect OSCC prognosis.

First, we examined the immunological properties of tumor‐infiltrating immune cells. Regarding immune cells and clinicopathological factors, multiple T‐cell subsets and CD20 positive cells tended to be less common in advanced stages and invasive phenotypes. Previous reports have revealed that an early tumor stage is associated with high infiltration of CD3 and CD8 positive T cells in HNSCC.^30^ Our results suggested that the immunological properties in the tumor microenvironment becomes immunosuppressive as a result of the acquisition of immune escape ability by cancer as it progresses. Macrophages infiltrating tumors, termed tumor‐associated macrophages (TAMs), are polarized into two types: M1 macrophages, which are inflammatory and have anti‐tumor activity, and M2 macrophages, which are anti‐inflammatory and characterized by anti‐tumor immunosuppression.^31^ The present study showed that CD163‐positive cells were more common in the advanced stages, suggesting that M2 macrophages are increased in the advanced stages. M2 TAMs are involved in the formation of an immunosuppressive microenvironment in advanced cancer,^32^ and the present results with CD163‐positive cells confirmed previous findings.

Next, we examined the expression of PD‐L1, a ligand for the representative immune checkpoint molecule PD‐1, in tumor and immune cells as factors determining the immunological properties of OSCC in the tumor microenvironment. PD‐L1 is used for the complementary diagnosis of anti‐PD‐1 inhibitor therapy.

Regarding PD‐L1 status and immune cells, PD‐L1‐positive cases were associated with abundant immune cell infiltrates, and PD‐L1 expression in immune cells was strongly correlated with all immune cell infiltrates. Kowanetz et al. stated that the PD‐L1 expression in immune cells is the result of an adaptive immune resistance mechanism mediated by IFN‐γ produced by TILs.^33^ Our results also suggest that pre‐existing inflammation in OSCC creates an inflammatory microenvironment that enhances PD‐L1 expression.

Survival analysis of PD‐L1 showed that PD‐L1 expression in tumor cells was not associated with prognosis, whereas that in immune cells was associated with significantly prolonged RFS. These results are consistent with that by Kim et al. that PD‐L1 in tumor and immune cells is independently regulated and may be involved in immunosuppression and prognosis through different mechanisms.^18^ Miranda‐Galvis M et.al demonstrated that high PD‐L1 expression in tumor cells was not a significant prognostic factor for disease free survival and OS, but a reactive patchy PD‐L1 pattern was associated worse OS.^34^ This immune reactive pattern may be helpful for understanding the role of PD‐L1 in tumor and immune cells in OSCC.

Previous studies have shown that effector T cells expressing PD‐L1 under IFN‐γ stimulation may be autoinhibited via the PD‐L1/PD‐1 mechanism.^35^ However, it is unclear which PD‐L1‐expressing effector T cells interact with which PD‐1 positive cells. We believe that it is important to clarify the role of PD‐L1 in immune cells.

Furthermore, we reported, for the first time, the relationship between SP categories and tumor‐infiltrating immune cells in OSCC. SP, a DR specific to OSCC, is classified into inflammatory, mature, intermediate, and immature.^20^ DR refers to an abnormal proliferation of fibrous connective tissue around cancer cell nests formed by the degradation and remodeling of the extracellular matrix by CAF.^36^ This study showed that the inflammatory type had more positive cells for all immune cell markers than the immature type, especially CD3, CD4, and CD8, compared with the mature/intermediate type. In a preliminary study of a small number of OSCC cases, myeloperoxidase (MPO)‐positive cells, a marker of myeloid cells, were fewer than lymphocytes, and did not differ by SP category. These results suggest that the SP category in OSCC is strongly associated with T‐cell infiltration, which play an important role in anti‐tumor immune responses. The tumor microenvironment has been proposed to be classified into three phenotypes according to immunological characteristics: inflamed, excluded, and immune desert.^37^ Inflamed tumor microenvironments are characterized by abundant immune cell infiltration (i.e., hot tumors), while immune deserts (i.e., cold tumors) are characterized by a lack of T‐cell infiltration. We consider that the “Inflammatory” type of SP corresponds to the inflamed immune phenotype, and the “Immature” type to the immune desert. We also speculate that the lower percentage of PD‐L1 IC‐positive cases and lower GBP5 stroma scores in the immature type were caused by reduced immune cell infiltration, that is, a lack of immune response in the tumor stroma. In HNSCC, CAFs regulates transforming growth factor beta (TGFβ) and is involved in suppressing T cell function.^38^ Another study shows that a specific subtype of CAFs can release TGFβ‐dependent immunosuppression.^39^ These results suggest that SP provide information on the histological phenotypes of multiple subsets of CAFs with different immunological functions. Thus, SP may be a useful prognostic indicator of OSCC, reflecting both the invasive and immunomodulatory potential of CAFs. Further research is needed to determine whether SP represent the entire immune microenvironment.

In this study, transcriptome analysis of the TCGA and GEO datasets identified seven immune‐related genes specific to OSCC. Based on our preliminary analysis, GBP5 was selected among these genes as a novel candidate immune‐related biomarker and subjected to histopathological examination.

GBP5 is a member of the IFN‐γ‐inducible GTPase subfamily and plays an important role in the activation of the nucleotide‐binding oligomerization domain‐like receptor family pyrin domain containing 3 (NLRP3) inflammasome in response to specific signals from pathogens and other inflammasome priming agents. GBP5 primarily contributes to inflammation and macrophage activation in the innate immune system.^40^, ^41^ Moreover, its possible involvement in tumor immunity has recently been reported.^42^ First, we investigated the association between GBP5, immune cell infiltration of tumors, and PD‐L1 expression. Our results showed that high GBP5 scores in tumors and stroma correlated with high infiltration of all immune cells and high expression of PD‐L1 in both tumor and immune cells. These results lead us to speculate that IFN‐γ upregulated by tumor immune responses enhances the expression of GBP5 and simultaneously induces the PD‐L1 expression and immune cell infiltration. Our finding is consistent with those of others showing that GBP5 expressions correlates with immune cell infiltration in gastric adenocarcinomas^27^ and other cancers^42^; thus supporting the possibility that GBP5 may be used as a biomarker to predict the therapeutic effect of ICIs.^42^, ^43^

To verify the significance of GBP5 as a prognostic factor, we examined the relationship between GBP5 expression and the prognosis of tumors and stroma. To the best of our knowledge, this is the first report on such an investigation. This study showed that GBP5 expression in the tumor and stroma did not affect survival; however, patients with low GBP5 expression in the tumor and high GBP5 expression in the stroma had a better prognosis than other patients. In previous HNSCC reports, GBP5 expression was not associated with survival. However, in OSCC reports, it was associated with poor prognosis in subgroups with lymph node metastasis and moderate/poor tumor grade.^28^, ^44^ Notably, previous studies on prognosis were based on TCGA database analyses without considering the localization of GBP5 expression. Our results suggest that the function of GBP5 in the tumor microenvironment may differ from that in the stroma.

Regarding molecular mechanisms of GBP5 in relation to tumors, GBP5 has been shown to enhance tumor cell invasive potential via activation of IFN‐γ/signal transducer and activator of transcription 1 (STAT1) and TNF‐α/nuclear factor‐kappa B (NF‐κB) signaling cascades or Janus kinase 1 (JAK1)‐STAT1/GBP5/CXCL8 feedback loop in cancers other than head and neck.^45^, ^46^ Although GBP5 has been reported to be involved in cell proliferation, tumor invasion, migration, epithelial‐mesenchymal transition (EMT), and cancer stem cell maintenance in OSCC,^28^ the molecular mechanism is still unclear.

IFN‐γ, which regulates GBP5, is important for the development and differentiation of immune cells involved in anti‐tumor immune responses and immune editing, generally acting in a tumor suppressive manner in the tumor microenvironment.^43^ The function of IFN‐γ depends on the responding cells, the surrounding cytokine environment, or the duration of IFN‐γ signaling, and prolonged IFN‐γ signaling has been shown to act on tumorigenesis via immunosuppression and angiogenesis.^35^ The oral cavity is prone to chronic inflammation, known as periodontal disease, due to infection by diverse bacterial flora.^46^ A recent paper proposed that polymorphic microbiomes in oral cavity are implicated in modulating tumor phenotypes.^47^

Although the association of oral bacteria with chronic inflammation and cancer has been reported, it is unclear whether inflammatory mediators are important for tumor development and growth whether they create a microenvironment that is permissive for cancer progression.^48^ Future studies to clarify how GBP5 functions in OSCC proliferation, invasion, and tumor immune mechanisms will provide new insights into a series of paradigms of chronic inflammation and carcinogenesis.

Finally, the discussion is subject to several limitations inherent to our study. The first is a selection bias because this was a single‐center, retrospective, observational study. In our hospital, neoadjuvant chemotherapy is sometimes administrated to patients with large tumors (cT4) or extranodal extensions that are predicted to have poor outcomes. These cases were excluded from this study, which may have led to biased patient characteristics. The second limitation is the lack of standardized cut‐off values. In this study, we used the median cutoff value for binarization of continuous variables and 5% for PD‐L1, which has been frequently used in previous studies. Third, this study was based solely on database analysis and immunohistochemical staining. Therefore, the functional significance and causal relationships of each cell and molecule could not be determined from the results of this study. In vivo and in vitro studies are required to clarify these issues.

## 5 CONCLUSIONS

We found the following in relation to the immunological properties of the tumor microenvironment in OSCC: Stromal pattern may be a prognostic factor, reflecting both the invasive potential and immunomodulatory capacity of CAFs; GBP5 correlates with PD‐L1 expression and immune cell infiltration and is associated with OSCC survival. We believe that GBP5 is a potential biomarker for predicting prognosis or therapeutic response to ICIs. Future studies to elucidate the molecular mechanisms by which GBP5 affects the immunological tumor microenvironment may provide new therapeutic approaches for OSCC.

## AUTHOR CONTRIBUTIONS

**Masayo Hasegawa:** Conceptualization (equal); data curation (lead); investigation (equal); methodology (lead); writing – original draft (lead). **Yusuke Amano:** Funding acquisition (lead); investigation (equal); writing – original draft (supporting). **Atsushi Kihara:** Formal analysis (equal); visualization (equal). **Daisuke Matsubara:** Resources (supporting); validation (lead). **Noriyoshi Fukushima:** Project administration (supporting); supervision (equal). **Hideyuki Takahashi:** Formal analysis (equal); methodology (supporting). **Kazuaki Chikamatsu:** Formal analysis (equal); validation (supporting). **Hiroshi Nishino:** Resources (equal); writing – original draft (supporting). **Yoshiyuki Mori:** Resources (equal); writing – original draft (supporting). **Naohiro Yoshida:** Supervision (equal); writing – review and editing (supporting). **Toshiro Niki:** Funding acquisition (supporting); project administration (lead); writing – review and editing (supporting).

## FUNDING INFORMATION

This work was supported in part by the Japan Society for the Promotion of Science KAKENHI (grant number 19K10094 to YA).

## CONFLICT OF INTEREST STATEMENT

The authors declare no conflicts of interest associated with this manuscript.

## ETHICS STATEMENT

This study was approved by the Ethics Review Committee of Jichi Medical University (approval no: Rin 22–012) and was conducted in accordance with the Declaration of Helsinki. TCGA and GEO databases are open access and available to the public.

## CONSENT STATEMENT

Consent was obtained from all the patients using an opt‐out method, instead of written consent. Explanatory documents were posted on the hospital's website.

## Supporting information


**Figure S1.** Representative figures of immunohistochemistry showing high and low numbers of tumor‐infiltrating immune cells (original magnification: ×200).

**Figure S2.** Representative images of PD‐L1 expression in tumor cells and immune cells by immunohistochemistry (original magnification: ×200). Abbreviations: PD‐L1, programmed death‐ligand 1.

**Figure S3.** Kaplan–Meier curves of overall and recurrence‐free survival in the 110 patients with OSCC cohort. Abbreviations: OSCC, oral squamous cell carcinoma.

**Figure S4.** Correlation between PD‐L1 expression and the patient prognosis. Kaplan–Meier curves showing the (a, b) overall and (c, d) recurrence‐free survival of patients with OSCC according to PD‐L1 expression. HR and 95% CI were indicated in the figures. *p*‐values were determined using log‐rank tests. (a, c) Survival data comparing the two groups with a cutoff value of TC 5%. (b, d) Survival data comparing the two groups with a cutoff value of IC 5%. Abbreviations: PD‐L1, programmed death‐ligand 1; OSCC, oral squamous cell carcinoma; HR, hazard ratios; CI, confidence intervals; TC, PD‐L1 expression in tumor cells; IC, PD‐L1 expression in immune cells.

Table S1:

Table S2:

Table S3:

Table S4:

Table S5:

Table S6:

## Data Availability

Some of the results presented in this manuscript are based on data generated by TCGA and GEO databases.
