# Supplementary figures and images for: Guanylate binding protein 5 is an immune‐related biomarker of oral squamous cell carcinoma: A retrospective prognostic study with bioinformatic analysis
Source: Cancer Med. 2024 Jul 8;13(13):e7431. doi: 10.1002/cam4.7431 (PMC11231040; doi:10.1002/cam4.7431)

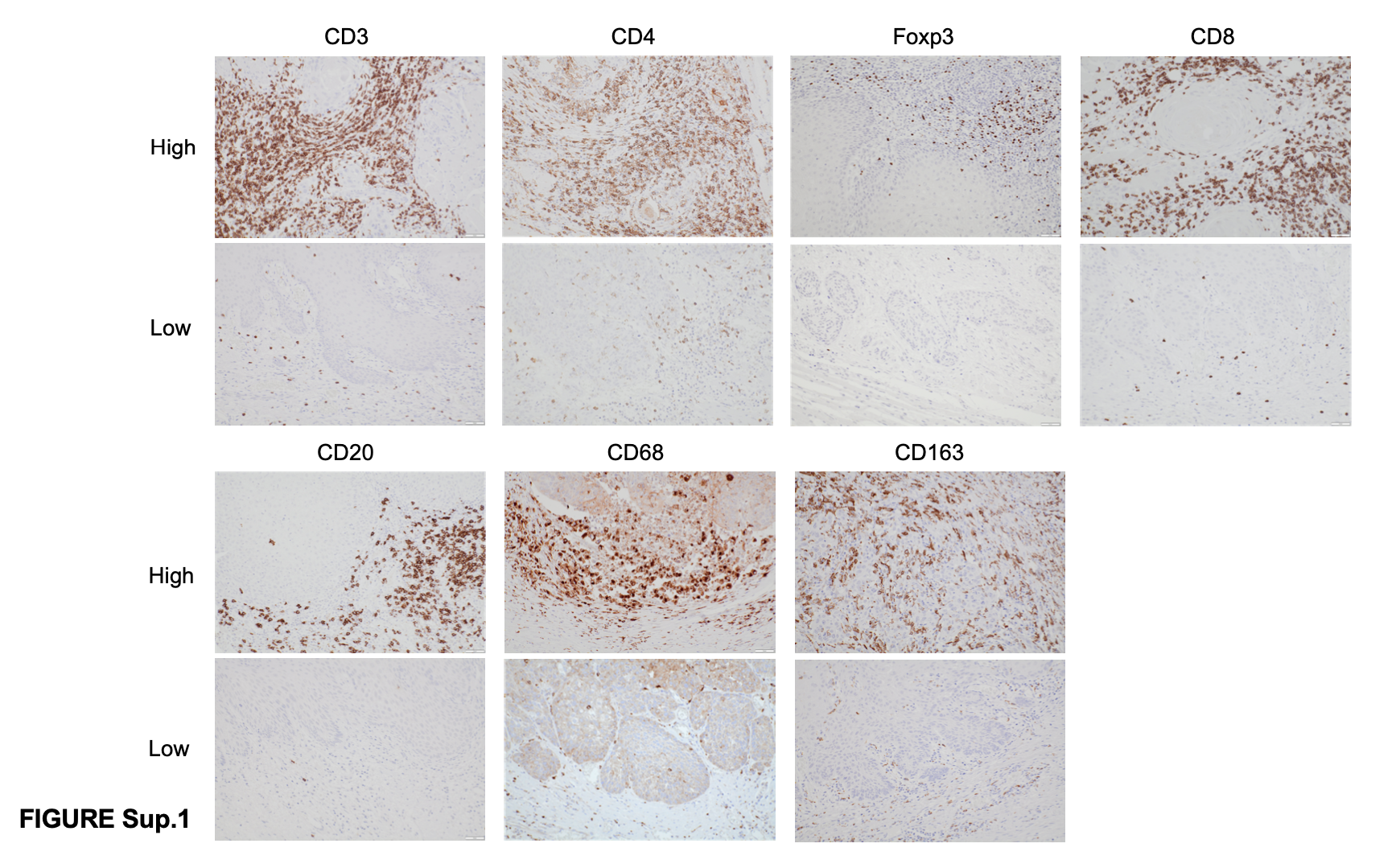

Supplement: Supplementary file 1 — Figure S1. Representative figures of immunohistochemistry showing high and low numbers of tumor‐infiltrating immune cells (original magnification: ×200). [file CAM4-13-e7431-s008.tiff]

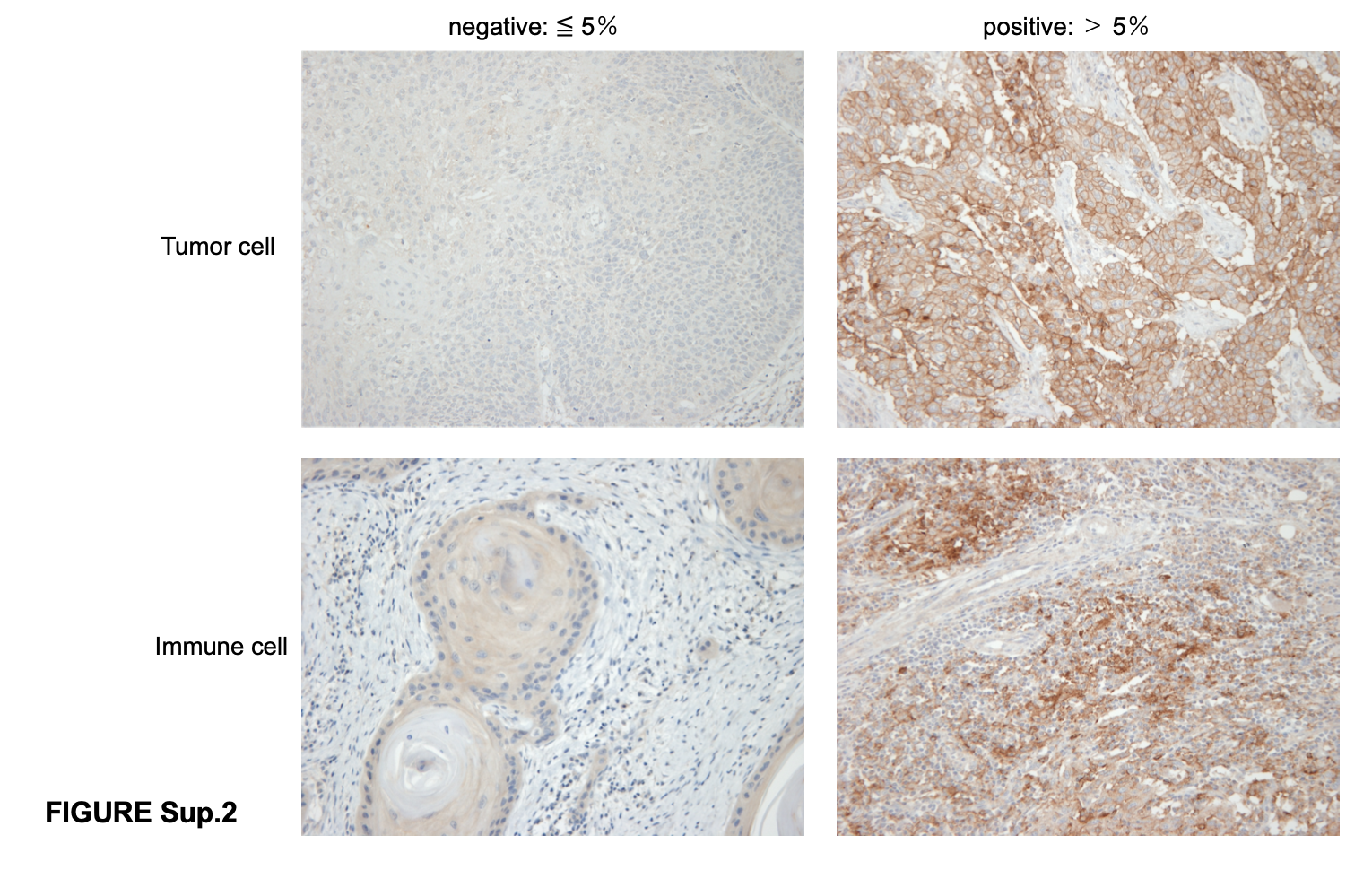

Supplement: Supplementary file 2 — Figure S2. Representative images of PD‐L1 expression in tumor cells and immune cells by immunohistochemistry (original magnification: ×200). Abbreviations: PD‐L1, programmed death‐ligand 1. [file CAM4-13-e7431-s010.tiff]

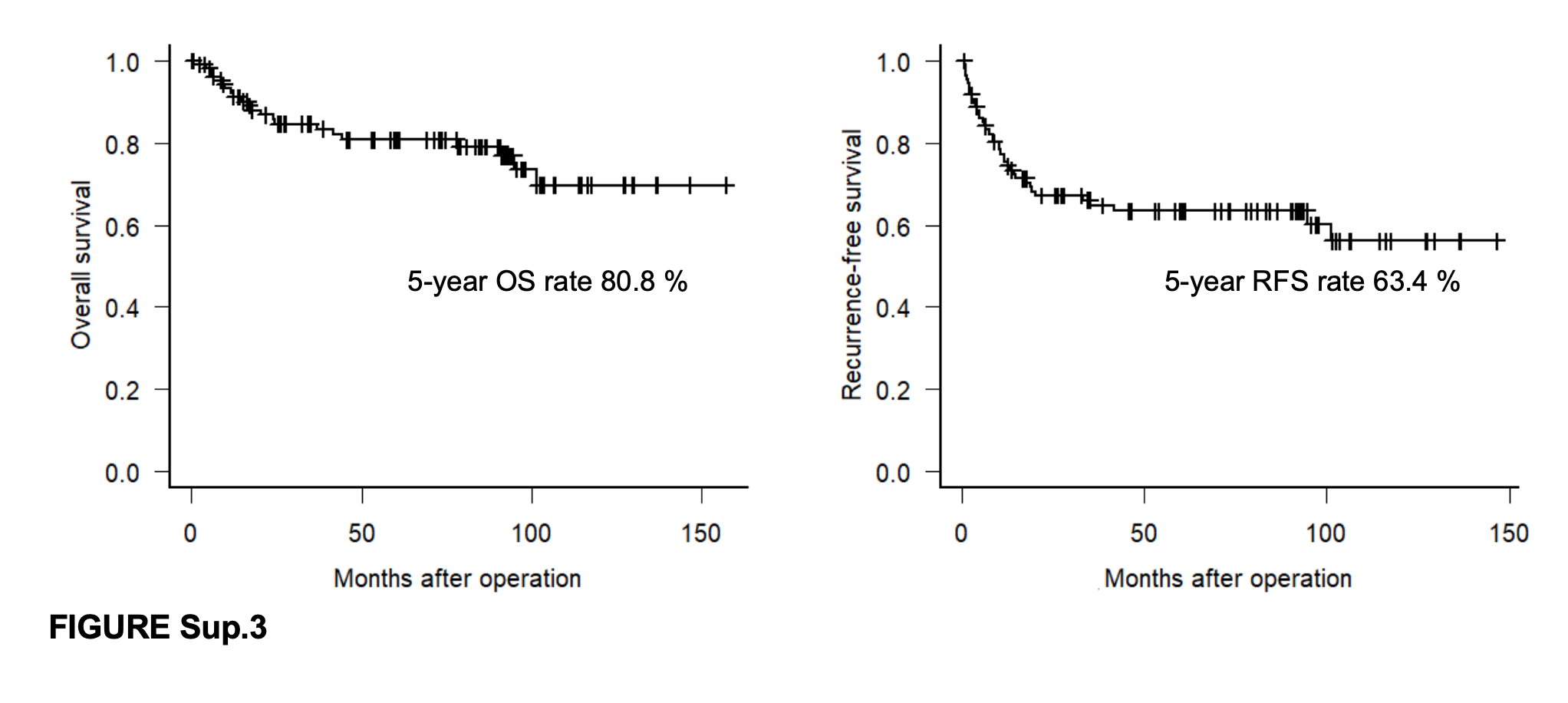

Supplement: Supplementary file 3 — Figure S3. Kaplan–Meier curves of overall and recurrence‐free survival in the 110 patients with OSCC cohort. Abbreviations: OSCC, oral squamous cell carcinoma. [file CAM4-13-e7431-s002.tiff]

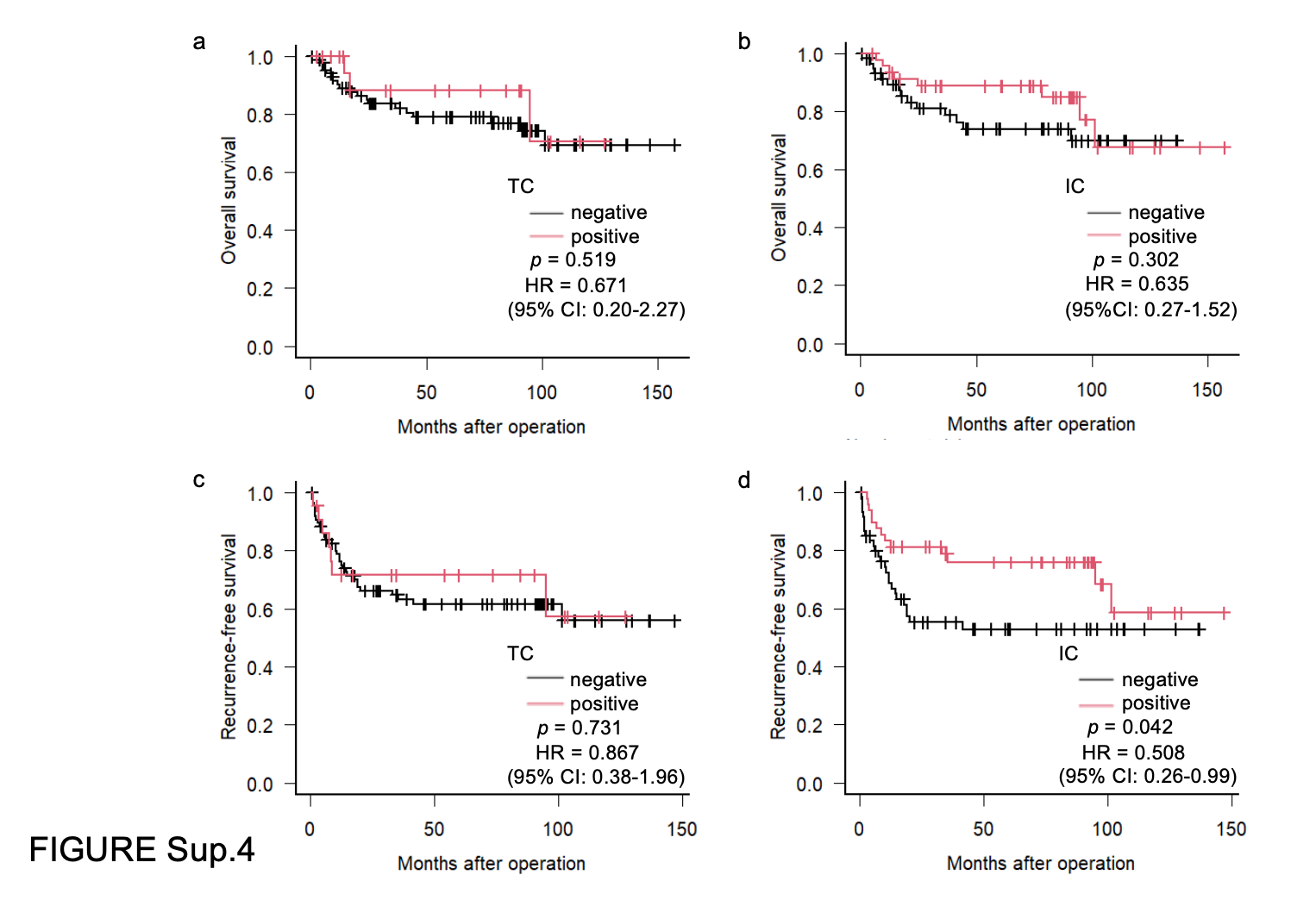

Supplement: Supplementary file 4 — Figure S4. Correlation between PD‐L1 expression and the patient prognosis. Kaplan–Meier curves showing the (a, b) overall and (c, d) recurrence‐free survival of patients with OSCC according to PD‐L1 expression. HR and 95% CI were indicated in the figures. p‐values were determined using log‐rank tests. (a, c) Survival data comparing the two groups with a cutoff value of TC 5%. (b, d) Survival data comparing the two groups with a cutoff value of IC 5%. Abbreviations: PD‐L1, programmed death‐ligand 1; OSCC, oral squamous cell carcinoma; HR, hazard ratios; CI, confidence intervals; TC, PD‐L1 expression in tumor cells; IC, PD‐L1 expression in immune cells. [file CAM4-13-e7431-s007.tiff]
