# Supplementary material for: Guanylate binding protein 5 is an immune‐related biomarker of oral squamous cell carcinoma: A retrospective prognostic study with bioinformatic analysis
Source: Cancer Med. 2024 Jul 8;13(13):e7431. doi: 10.1002/cam4.7431 (PMC11231040; doi:10.1002/cam4.7431)
Supplement: Supplementary file 5 — Table S1: [file CAM4-13-e7431-s004.docx]

Table S1 Mean, median and range for different immune cells (CD3+, CD4+, Foxp3+, CD8+, CD20+, CD68+, CD163+) within OSCC

| **Marker** | Mean ± SD | Median (range) |
| --- | --- | --- |
| **CD3** | 558.6 ± 305.2 | 552.2 (40.6 - 1375.0) |
| **CD4** | 265.4 ± 135.7 | 249.9 (20.4 - 605.2) |
| **Foxp3** | 94.9 ± 55.6 | 83.4 (5.0 - 252.6) |
| **CD8** | 268.8 ± 176.5 | 247.4 (10.8 - 916.0) |
| **CD20** | 77.8 ± 78.2 | 55.6 (0 - 406.2) |
| **CD68** | 137.8 ± 70.6 | 131.0 (4.2 - 310.6) |
| **CD163** | 91.6 ± 52.4 | 88.4 (4.8 - 285.4) |

Abbreviations: SD, standard deviation.
