# Supplementary material for: Guanylate binding protein 5 is an immune‐related biomarker of oral squamous cell carcinoma: A retrospective prognostic study with bioinformatic analysis
Source: Cancer Med. 2024 Jul 8;13(13):e7431. doi: 10.1002/cam4.7431 (PMC11231040; doi:10.1002/cam4.7431)
Supplement: Supplementary file 6 — Table S2: [file CAM4-13-e7431-s001.docx]

Table S2　Association between tumor-infiltrating immune cells and clinicopathological factors

| **Parameters** | **Case number  n(%)** | **CD3 (mean)** | ***p*** | **CD4 (mean)** | ***p*** | **Foxp3 (mean)** | ***p*** | **CD8 (mean)** | ***p*** | **CD20 (mean)** | ***p*** | **CD68 (mean)** | ***p*** | **CD163 (mean)** | ***p*** |
| --- | --- | --- | --- | --- | --- | --- | --- | --- | --- | --- | --- | --- | --- | --- | --- |
| **Sex** |  |  |  |  |  |  |  |  |  |  |  |  |  |  |  |
| Male | 61(55.45) | 515.93 | 0.102 | 246.157 | 0.097 | 85.67 | 0.005** | 252.55 | 0.283 | 73.65 | 0.538 | 128.16 | 0.112 | 89.08 | 0.569 |
| Female | 49(44.55) | 611.72 |  | 289.41 |  | 106.47 |  | 289.04 |  | 82.93 |  | 149.74 |  | 94.84 |  |
| **Age (years)** |  |  |  |  |  |  |  |  |  |  |  |  |  |  |  |
| ＜65 | 47(42.73) | 506.97 | 0.126 | 241.03 | 0.104 | 81.05 | 0.023* | 245.70 | 0.237 | 78.80 | 0.907 | 127.83 | 0.204 | 78.65 | 0.002** |
| ≧65 | 63(57.27) | 597.11 |  | 283.63 |  | 105.29 |  | 286.04 |  | 77.03 |  | 145.19 |  | 101.34 |  |
| **Location** |  |  |  |  |  |  |  |  |  |  |  |  |  |  |  |
| Tongue | 83(75.45) | 565.60 | 0.675 | 268.85 | 0.644 | 96.31 | 0.650 | 271.90 | 0.748 | 76.58 | 0.778 | 135.71 | 0.593 | 87.62 | 0.236 |
| Others | 27(24.55) | 537.09 |  | 254.88 |  | 90.70 |  | 259.28 |  | 81.49 |  | 144.12 |  | 104.02 |  |
| **pT status** |  |  |  |  |  |  |  |  |  |  |  |  |  |  |  |
| Tis–2 | 80(72.73) | 621.79 | 0.000*** | 294.62 | 0.000*** | 108.79 | 0.000*** | 299.57 | 0.003** | 93.85 | 0.000*** | 139.81 | 0.624 | 89.43 | 0.471 |
| 3–4 | 30(27.27) | 390.11 |  | 187.58 |  | 57.99 |  | 186.77 |  | 34.93 |  | 132.35 |  | 97.56 |  |
| **pN status** |  |  |  |  |  |  |  |  |  |  |  |  |  |  |  |
| 0 | 80(72.73) | 587.91 | 0.100 | 279.52 | 0.075 | 103.25 | 0.010** | 280.26 | 0.268 | 85.84 | 0.077 | 137.02 | 0.856 | 88.61 | 0.324 |
| 1–3 | 30(27.27) | 480.44 |  | 227.84 |  | 72.75 |  | 238.26 |  | 56.30 |  | 139.78 |  | 99.73 |  |
| **Grade** |  |  |  |  |  |  |  |  |  |  |  |  |  |  |  |
| 1 | 67(60.91) | 583.52 | 0.287 | 279.08 | 0.189 | 101.10 | 0.175 | 275.67 | 0.612 | 86.87 | 0.128 | 154.31 | 0.002** | 101.04 | 0.002** |
| 2–3 | 43(39.09) | 519.77 |  | 244.15 |  | 85.32 |  | 258.10 |  | 63.62 |  | 112.01 |  | 77.00 |  |
| **pStage** |  |  |  |  |  |  |  |  |  |  |  |  |  |  |  |
| 0–Ⅱ | 70(63.64) | 603.51 | 0.041* | 286.92 | 0.027* | 108.02 | 0.000*** | 287.11 | 0.151 | 91.03 | 0.018* | 135.51 | 0.658 | 84.77 | 0.007** |
| Ⅲ–Ⅳ | 40(36.36) | 480.02 |  | 227.81 |  | 72.04 |  | 236.76 |  | 54.60 |  | 141.74 |  | 103.68 |  |
| **YK** |  |  |  |  |  |  |  |  |  |  |  |  |  |  |  |
| 1–3 | 50(45.45) | 624.59 | 0.102 | 294.58 | 0.082 | 104.95 | 0.118 | 296.04 | 0.301 | 97.61 | 0.052 | 150.19 | 0.415 | 97.26 | 0.783 |
| 4 | 52(47.27) | 525.73 |  | 248.10 |  | 87.93 |  | 259.50 |  | 67.12 |  | 139.23 |  | 94.44 |  |
| **Ly invasion** |  |  |  |  |  |  |  |  |  |  |  |  |  |  |  |
| Negative | 70(63.64) | 586.12 | 0.212 | 282.53 | 0.080 | 106.13 | 0.005** | 276.05 | 0.571 | 85.03 | 0.200 | 133.89 | 0.449 | 86.82 | 0.203 |
| Positive | 40(36.36) | 510.45 |  | 235.49 |  | 75.34 |  | 256.12 |  | 65.10 |  | 144.56 |  | 100.09 |  |
| **V invasion** |  |  |  |  |  |  |  |  |  |  |  |  |  |  |  |
| Negative | 52(47.27) | 601.32 | 0.166 | 292.65 | 0.046* | 109.50 | 0.009** | 286.69 | 0.317 | 89.93 | 0.123 | 137.49 | 0.968 | 85.27 | 0.229 |
| Positive | 58(52.73) | 520.30 |  | 241.02 |  | 81.88 |  | 252.77 |  | 66.89 |  | 138.03 |  | 97.36 |  |
| **Neu invasion** |  |  |  |  |  |  |  |  |  |  |  |  |  |  |  |
| Negative | 75(68.18) | 598.86 | 0.042* | 288.48 | 0.008** | 103.95 | 0.012* | 289.53 | 0.071 | 85.77 | 0.117 | 136.53 | 0.789 | 88.56 | 0.369 |
| Positive | 35(31.82) | 472.32 |  | 216.02 |  | 75.61 |  | 224.40 |  | 60.68 |  | 140.43 |  | 98.25 |  |
| **Depth** |  |  |  |  |  |  |  |  |  |  |  |  |  |  |  |
| ＜10 | 78(76.47) | 610.78 | 0.028* | 289.01 | 0.014* | 104.62 | 0.005** | 294.62 | 0.077 | 95.10 | 0.002** | 144.04 | 0.880 | 92.21 | 0.201 |
| ≧10 | 24(23.53) | 455.26 |  | 211.98 |  | 69.15 |  | 221.48 |  | 39.72 |  | 146.44 |  | 107.57 |  |
| **SP** |  |  |  |  |  |  |  |  |  |  |  |  |  |  |  |
| Inf | 35(34.31) | 713.51 | 0.002** | 340.00 | 0.000*** | 118.73 | 0.004** | 349.65 | 0.011* | 113.35 | 0.006** | 173.67 | 0.001** | 115.74 | 0.014* |
| Mat/Int | 40(39.22) | 531.37 | 0.023^a^ | 253.74 | 0.011^a^ | 92.21 |  | 243.50 | 0.026^a^ | 74.98 |  | 140.51 |  | 88.51 |  |
| Imm | 27(26.47) | 457.03 | 0.002^b^ | 206.68 | 0.000^b^ | 73.19 | 0.003^b^ | 234.03 | 0.029^b^ | 52.01 | 0.007^b^ | 112.99 | 0.001^b^ | 80.84 | 0.021^b^ |

**p* ＜ 0.05, ** *p* ＜ 0.01, *** *p* ＜ 0.001, ^a^ inflammatory type vs. mature/intermediate type (*p* ＜ 0.05 after Bonferroni correction), ^b^ inflammatory type vs. immature type (*p* ＜ 0.05 after Bonferroni correction)

Abbreviations: YK, Yamamoto–Kohama; SP, stromal pattern; Inf, inflammatory; Mat, mature; Int, intermediate; Imm, immature
