# Supplementary material for: Guanylate binding protein 5 is an immune‐related biomarker of oral squamous cell carcinoma: A retrospective prognostic study with bioinformatic analysis
Source: Cancer Med. 2024 Jul 8;13(13):e7431. doi: 10.1002/cam4.7431 (PMC11231040; doi:10.1002/cam4.7431)
Supplement: Supplementary file 7 — Table S3: [file CAM4-13-e7431-s006.docx]

Table S3　Correlations between PD-L1 expression and clinicopathological parameters

| **Parameters** | **Case**  **n (%)** | **TC** |  |  | **IC** |  |  |
| --- | --- | --- | --- | --- | --- | --- | --- |
|  |  | **negative**  **n (%)** | **positive**  **n (%)** | ***p*** | **negative**  **n (%)** | **positive**  **n (%)** | ***p*** |
| **Sex** |  |  |  |  |  |  |  |
| Male | 61 (55.45) | 53 (60.2) | 8 (36.4) | 0.056 | 39 (62.9) | 22 (45.8) | 0.085 |
| Female | 49 (44.55) | 35 (39.8) | 14 (64.6) |  | 23 (37.1) | 26 (54.2) |  |
| **Age (years)** |  |  |  |  |  |  |  |
| ＜65 | 47 (42.73) | 38 (43.2) | 9 (40.9) | 1.000 | 28 (45.2) | 19 (39.6) | 0.567 |
| ≧65 | 63 (57.27) | 50 (56.8) | 13 (59.8) |  | 34 (54.2) | 29 (60.4) |  |
| **Location** |  |  |  |  |  |  |  |
| Tongue | 83 (75.45) | 67 (76.1) | 16 (72.3) | 0.784 | 45 (72.6) | 38 (79.2) | 0.506 |
| Others | 27 (24.55) | 21 (23.9) | 6 (27.7) |  | 17 (27.4) | 10 (20.8) |  |
| **pT status** |  |  |  |  |  |  |  |
| Tis–2 | 80 (72.73) | 63 (71.6) | 17 (77.3) | 0.790 | 42 (67.7) | 38 (79.2) | 0.202 |
| 3–4 | 30 (27.27) | 25 (28.4) | 5 (22.7) |  | 20 (32.3) | 10 (20.8) |  |
| **pN status** |  |  |  |  |  |  |  |
| 0 | 80 (72.73) | 63 (71.6) | 17 (77.3) | 0.790 | 42 (67.7) | 38 (79.2) | 0.202 |
| 1–3 | 30 (27.27) | 25 (28.4) | 5 (22.7) |  | 20 (32.3) | 10 (20.8) |  |
| **Grade** |  |  |  |  |  |  |  |
| 1 | 67 (60.91) | 54 (61.4) | 13 (59.1) | 1.000 | 33 (53.2) | 34 (70.8) | 0.077 |
| 2–3 | 43 (39.09) | 34 (38.6) | 9 (40.9) |  | 29 (46.8) | 14 (29.2) |  |
| **pStage** |  |  |  |  |  |  |  |
| 0–Ⅱ | 70 (63.64) | 56 (63.4) | 14 (63.4) | 1.000 | 37 (59.7) | 33 (68.8) | 0.424 |
| Ⅲ–Ⅳ | 40 (36.36) | 32 (36.4) | 8 (36.4) |  | 25 (40.3) | 15 (31.2) |  |
| **YK** |  |  |  |  |  |  |  |
| 1–3 | 50 (45.45) | 36 (45.0) | 14(63.6) | 0.151 | 23(41.1) | 27 (58.7) | 0.111 |
| 4 | 52 (47.27) | 44 (55.0) | 8(36.4) |  | 33(58.9) | 19 (41.3) |  |
| **Ly invasion** |  |  |  |  |  |  |  |
| Negative | 70 (63.64) | 54 (61.4) | 16 (72.7) | 0.458 | 38 (61.3) | 32 (66.7) | 0.690 |
| Positive | 40 (36.36) | 34 (38.6) | 6 (27.3) |  | 24 (38.7) | 16 (33.3) |  |
| **V invasion** |  |  |  |  |  |  |  |
| Negative | 52 (47.27) | 38 (43.2) | 14 (63.6) | 0.099 | 28 (45.2) | 24 (50.0) | 0.701 |
| Positive | 58 (52.73) | 50 (56.8) | 8 (36.4) |  | 34 (54.8) | 24 (50.0) |  |
| **Neu invasion** |  |  |  |  |  |  |  |
| Negative | 75 (68.18) | 58 (65.9) | 17 (77.3) | 0.443 | 44 (71.0) | 31 (64.6) | 0.538 |
| Positive | 35 (31.82) | 30 (34.1) | 5 (22.7) |  | 18 (29.0) | 17 (35.4) |  |
| **Depth** |  |  |  |  |  |  |  |
| ＜10 | 78 (76.47) | 60 (75.0) | 18 (81.8) | 0.583 | 42 (75.0) | 36 (78.3) | 0.816 |
| ≧10 | 24 (23.53) | 20 (25.0) | 48 (12.2) |  | 14 (25.0) | 10 (21.7) |  |
| **SP** |  |  |  |  |  |  |  |
| Inf/Mat/Int | 75 (73.53) | 58 (72.5) | 17 (77.3) | 0.788 | 36 (64.3) | 39 (84.8) | 0.024* |
| Imm | 27 (26.47) | 22 (27.5) | 5 (22.7) |  | 20 (35.7) | 7 (15.2) |  |

**p* ＜ 0.05

Abbreviations: YK, Yamamoto–Kohama; SP, stromal pattern; Inf/Mat/Int, inflammatory/mature/intermediate; Imm, immature; TC, PD-L1 expression in tumor cells; IC, PD-L1 expression in immune cells.
