# Supplementary material for: Guanylate binding protein 5 is an immune‐related biomarker of oral squamous cell carcinoma: A retrospective prognostic study with bioinformatic analysis
Source: Cancer Med. 2024 Jul 8;13(13):e7431. doi: 10.1002/cam4.7431 (PMC11231040; doi:10.1002/cam4.7431)
Supplement: Supplementary file 8 — Table S4: [file CAM4-13-e7431-s009.docx]

Table S4　Correlations of tumor-infiltrating immune cells with PD-L1 expression in patients with OSCC

|  | **TC** |  |  |  | **IC** |  |  |  |
| --- | --- | --- | --- | --- | --- | --- | --- | --- |
| **Marker** |  | **mean** | **SD** | ***p*** |  | **mean** | **SD** | ***p*** |
| **CD3** |  |  |  |  |  |  |  |  |
|  | **negative** | 516.01 | 281.80 | 0.003** | **negative** | 437.24 | 247.25 | 0.000*** |
|  | **positive** | 728.95 | 341.30 |  | **positive** | 715.36 | 303.57 |  |
| **CD4** |  |  |  |  |  |  |  |  |
|  | **negative** | 249.29 | 131.00 | 0.012* | **negative** | 211.20 | 111.92 | 0.000*** |
|  | **positive** | 329.95 | 137.93 |  | **positive** | 335.46 | 132.49 |  |
| **CD8** |  |  |  |  |  |  |  |  |
|  | **negative** | 248.80 | 165.10 | 0.017* | **negative** | 214.65 | 150.79 | 0.000*** |
|  | **positive** | 348.84 | 200.85 |  | **positive** | 338.76 | 183.98 |  |
| **CD20** |  |  |  |  |  |  |  |  |
|  | **negative** | 73.40 | 77.97 | 0.241 | **negative** | 54.05 | 51.72 | 0.000*** |
|  | **positive** | 95.31 | 78.27 |  | **positive** | 108.43 | 94.85 |  |
| **CD68** |  |  |  |  |  |  |  |  |
|  | **negative** | 123.55 | 61.89 | 0.000*** | **negative** | 104.66 | 57.01 | 0.000*** |
|  | **positive** | 194.65 | 76.12 |  | **positive** | 180.54 | 63.56 |  |
| **CD163** |  |  |  |  |  |  |  |  |
|  | **negative** | 81.10 | 44.51 | 0.001*** | **negative** | 69.27 | 43.27 | 0.000*** |
|  | **positive** | 133.82 | 61.10 |  | **positive** | 120.54 | 49.32 |  |
| **Foxp3** |  |  |  |  |  |  |  |  |
|  | **negative** | 91.50 | 57.30 | 0.197 | **negative** | 72.59 | 46.56 | 0.000*** |
|  | **positive** | 108.65 | 46.69 |  | **positive** | 123.79 | 53.28 |  |

**p* ＜ 0.05 , ***p* ＜ 0.01, ****p* ＜ 0.001

Abbreviations: OSCC, oral squamous cell carcinoma; TC, PD-L1 expression in tumor cells; IC, PD-L1 expression in immune cells; SD, standard deviation.
