# Supplementary material for: Guanylate binding protein 5 is an immune‐related biomarker of oral squamous cell carcinoma: A retrospective prognostic study with bioinformatic analysis
Source: Cancer Med. 2024 Jul 8;13(13):e7431. doi: 10.1002/cam4.7431 (PMC11231040; doi:10.1002/cam4.7431)
Supplement: Supplementary file 9 — Table S5: [file CAM4-13-e7431-s005.docx]

Table S5　Association between clinicopathological parameters and patient survivals

|  | **OS** |  |  |  | **RFS** |  |  |  |
| --- | --- | --- | --- | --- | --- | --- | --- | --- |
| **Parameters** | **5-year**  **OS (%)** | ***p*** | **Hazard ratio** | **95%CI** | **5-year RFS (%)** | ***p*** | **Hazard ratio** | **95%CI** |
| **Sex** |  |  |  |  |  |  |  |  |
| Male | 82.6 | 0.692 | 0.844 | 0.364-1.957 | 63.3 | 0.792 | 1.089 | 0.577-2.057 |
| Female | 78.4 |  |  |  | 63.6 |  |  |  |
| **Age (years)** |  |  |  |  |  |  |  |  |
| ＜65 | 74.0 | 0.349 | 0.671 | 0.289-1.557 | 56.2 | 0.429 | 0.776 | 0.414-1.456 |
| ≧65 | 87.2 |  |  |  | 70.4 |  |  |  |
| **Location** |  |  |  |  |  |  |  |  |
| Tongue | 70.7 | 0.089 | 0.484 | 0.206-1.138 | 64.3 | 0.526 | 0.798 | 0.396-1.608 |
| Others | 84.3 |  |  |  | 61.0 |  |  |  |
| **pT status** |  |  |  |  |  |  |  |  |
| Tis–2 | 90.2 | 0.002** | 3.583 | 1.539-8.343 | 69.0 | 0.074 | 1.812 | 0.9356-3.51 |
| 3–4 | 53.0 |  |  |  | 46.8 |  |  |  |
| **pN status** |  |  |  |  |  |  |  |  |
| 0 | 89.5 | 0.004** | 3.248 | 1.403-7.515 | 72.4 | 0.001 | 2.707 | 1.427-5.134 |
| 1–3 | 58.3 |  |  |  | 40.0 |  |  |  |
| **Grade** |  |  |  |  |  |  |  |  |
| 1 | 72.0 | 0.249 | 0.614 | 0.266-1.418 | 71.5 | 0.137 | 0.622 | 0.331-1.169 |
| 2–3 | 86.8 |  |  |  | 51.5 |  |  |  |
| **pStage** |  |  |  |  |  |  |  |  |
| 0–Ⅱ | 93.5 | 0.000*** | 4.239 | 1.762-10.2 | 74.9 | 0.001** | 2.785 | 1.464-5.296 |
| Ⅲ–Ⅳ | 57.1 |  |  |  | 43.2 |  |  |  |
| **YK** |  |  |  |  |  |  |  |  |
| 1–3 | 91.4 | 0.069 | 2.254 | 0.916-5.534 | 79.6 | 0.007** | 2.458 | 1.244-4.857 |
| 4 | 68.7 |  |  |  | 44.7 |  |  |  |
| **Ly invasion** |  |  |  |  |  |  |  |  |
| Negative | 87.9 | 0.093 | 2.029 | 0.874-4.711 | 74.2 | 0.001** | 2.722 | 1.433-5.167 |
| Positive | 69.0 |  |  |  | 45.5 |  |  |  |
| **V invasion** |  |  |  |  |  |  |  |  |
| Negative | 95.5 | 0.005** | 3.972 | 1.433-11.01 | 80.2 | 0.002** | 2.896 | 1.425-5.886 |
| Positive | 68.1 |  |  |  | 49.1 |  |  |  |
| **Neu invasion** |  |  |  |  |  |  |  |  |
| Negative | 92.3 | 0.000*** | 5.370 | 2.163-13.33 | 75.2 | 0.000*** | 3.128 | 1.636-5.979 |
| Positive | 57.6 |  |  |  | 39.3 |  |  |  |
| **Depth** |  |  |  |  |  |  |  |  |
| ＜10 | 82.5 | 0.411 | 1.481 | 0.577-3.798 | 62.4 | 0.890 | 1.054 | 0.499-2.228 |
| ≧10 | 69.3 |  |  |  | 55.5 |  |  |  |
| **SP** |  |  |  |  |  |  |  |  |
| Inf/Mat/Int | 86.5 | 0.019* | 2.642 | 1.138-6.131 | 70.8 | 0.007** | 2.340 | 1.235-4.434 |
| Imm | 61.6 |  |  |  | 36.7 |  |  |  |

**p* ＜ 0.05, ***p* ＜ 0.01, ****p* ＜ 0.001. *P*-values were determined using the log-rank test.

Abbreviations: YK, Yamamoto–Kohama; SP, stromal pattern; Inf/Mat/Int, inflammatory/mature/intermediate; Imm, immature; OS, overall survival; RFS, recurrence-free survival; CI, confidence interval.
