# Supplementary material for: Guanylate binding protein 5 is an immune‐related biomarker of oral squamous cell carcinoma: A retrospective prognostic study with bioinformatic analysis
Source: Cancer Med. 2024 Jul 8;13(13):e7431. doi: 10.1002/cam4.7431 (PMC11231040; doi:10.1002/cam4.7431)
Supplement: Supplementary file 10 — Table S6: [file CAM4-13-e7431-s003.docx]

Table S6　Association between tumor-infiltrating immune cells and patient survivals

| **Marker** |  | **OS** |  |  |  |  | **RFS** |  |  |  |
| --- | --- | --- | --- | --- | --- | --- | --- | --- | --- | --- |
|  | **Cut off** | **5-yr OS (%)** | ***p*** | **Hazard ratio** | **95%CI** |  | **5-yr RFS (%)** | ***p*** | **Hazard ratio** | **95%CI** |
| **CD3 (n, %)** |  |  |  |  |  |  |  |  |  |  |
| Low  (55, 50%) | 552.2 | 72.7 | 0.256 | 0.613 | 0.261-1.439 |  | 64.3 | 0.629 | 1.168 | 0.621-2.196 |
| High  (55, 50%) |  | 72.7 |  |  |  |  | 62.9 |  |  |  |
| **CD4** |  |  |  |  |  |  |  |  |  |  |
| Low  (55, 50%) | 249.9 | 78.5 | 0.748 | 1.148 | 0.495-2.661 |  | 64.1 | 0.628 | 1.168 | 0.622-2.195 |
| High  (55, 50%) |  | 83.3 |  |  |  |  | 62.9 |  |  |  |
| **Foxp3** |  |  |  |  |  |  |  |  |  |  |
| Low  (55, 50%) | 83.4 | 75.0 | 0.643 | 0.820 | 0.354-1.902 |  | 61.8 | 0.983 | 1.007 | 0.537-1.889 |
| High  (55, 50%) |  | 86.0 |  |  |  |  | 64.9 |  |  |  |
| **CD8** |  |  |  |  |  |  |  |  |  |  |
| Low  (55, 50%) | 247.4 | 71.7 | 0.210 | 0.583 | 0.248-1.369 |  | 65.6 | 0.447 | 1.278 | 0.678-2.410 |
| High  (55, 50%) |  | 89.6 |  |  |  |  | 61.8 |  |  |  |
| **CD20** |  |  |  |  |  |  |  |  |  |  |
| Low  (55, 50%) | 55.6 | 75.6 | 0.763 | 0.879 | 0.381-2.029 |  | 67.0 | 0.348 | 1.356 | 0.716-2.568 |
| High  (55, 50%) |  | 85.8 |  |  |  |  | 60.1 |  |  |  |
| **CD68** |  |  |  |  |  |  |  |  |  |  |
| Low  (55, 50%) | 131.0 | 74.1 | 0.319 | 0.651 | 0.278-1.525 |  | 50.5 | 0.034* | 0.499 | 0.259-0.961 |
| High  (55, 50%) |  | 87.9 |  |  |  |  | 77.2 |  |  |  |
| **CD163** |  |  |  |  |  |  |  |  |  |  |
| Low  (55, 50%) | 88.4 | 80.6 | 0.846 | 1.086 | 0.471-2.508 |  | 58.5 | 0.582 | 0.837 | 0.445-1.578 |
| High  (55, 50%) |  | 81.5 |  |  |  |  | 69.6 |  |  |  |

**p* ＜ 0.05

*P*-values were determined using log-rank tests.

Abbreviations: OS, overall survival; RFS, recurrence-free survival; CI, confidence interval.
